# The Value of Hearing Aids for the Italian NHS: A Cost-utility Analysis

**DOI:** 10.1097/ONO.0000000000000018

**Published:** 2022-10-26

**Authors:** Giulia Fornaro, Patrizio Armeni, Andrea Albera, Michele Barbara

**Affiliations:** 1 Centre for Research on Health and Social Care Management, SDA Bocconi School of Management, Bocconi University, Milan, Italy; 2 Department of Social and Political Science, Bocconi University, Milan, Italy; 3 Dipartimento di Scienze Chirurgiche, Università degli Studi di Torino, Turin, Italy; 4 Department of Otorhinolaryngology, “Mons. Dimiccoli” Hospital, Barletta, Italy.

**Keywords:** Cost-utility analysis, Hearing aids, Hearing loss, Net monetary benefit analysis

## Abstract

**Objective:**

Hearing loss (HL) prevalence in Italy is expected to increase due to population aging. Hearing aids (HAs) are the main tool for HL rehabilitation; however, cost-utility analyses of HAs are limited. Our objective was to estimate the cost-utility of HAs use.

**Study Design:**

Cost-utility analysis.

**Setting:**

Italian National Healthcare Service, societal perspective.

**Patients, Intervention(s), and Main Outcome Measure(s):**

A multistate Markov model was developed to model a cohort of 55-year-old individuals starting from normal hearing and moving across HL states to compare cost-utility and net monetary benefit of HA use accompanied by post-purchase service, HA use alone, and no treatment. Parameters were estimated using secondary data. Incremental cost-utility ratio (ICUR) and incremental net monetary benefit (INMB) were computed against a €16,625/quality-adjusted life year (QALY) willingness-to-pay (WTP) threshold. Deterministic and probabilistic sensitivity analysis (DSA, PSA) was implemented to assess how uncertainty affected results. Scenario analysis was performed on different assumptions on costs, dropout and compliance rates.

**Results:**

The model suggests HAs use is a cost-effective strategy compared to no treatment (in the base case: incremental costs €429–€476, incremental QALY gain 0.18 and 0.19, ICUR €2‚404/QALY–€2‚450/QALY, INMB €2‚476–€2‚682 for male and female cohort, respectively). By assuming no dropout, INMBs increase up to €10,643–€10,728. DSA highlights that utility weights contribute the most to model uncertainty, PSA shows that the treatment has 97.8%–97.3% probability of being cost-effective at the WTP threshold considered.

**Conclusions:**

We proposed an original model to assess the cost-utility of HAs use; the application to the Italian setting suggests the treatment is cost-effective, reinforcing the importance of early uptake.

According to the most recent estimates available from Global Burden of Disease study, age-related hearing loss (HL) accounted for 1‚460 million cases worldwide in 2019 and was the fourth-ranked cause of years lived with disability in 2019 (1,2). The dominant cause is presbycusis, defined as the gradual loss of hearing with age. In Italy, it was estimated that almost 7 million people suffer from HL and prevalence tends to increase with aging, with a prevalence of 25% in individuals between 61 and 80 years of age and of 50% in individuals older than 80 years; as a consequence, prevalence is expected to increase over the next years due to population aging (3). Age-related HL is associated, among others, to a reduction in quality of life (QoL), higher susceptibility to social isolation (4), depression (5), and it is recognized as a risk factor for dementia (6,7). Hearing aids (HAs) are considered an effective rehabilitation instrument that can improve QoL (8) and also mitigate other detrimental effects of age-related HL, for example, limiting the excess risk for dementia (9). However, HAs uptake is still relatively limited and frequently characterized by underuse or abandonment (10); potential barriers could be, among others, the persistence of social stigma around HA wearers (10), costs, inadequate customization of the device. In the health economics and outcomes research literature, few examples of cost-utility analyses on HAs for age-related HL exist. The most relevant ones and related results are: the work by Boas et al (11) (incremental cost-utility ratio [ICUR] of €18,046/quality-adjusted life year [QALY] and €21,154/QALY, The Netherlands), the one by Joore et al (12) (€15,807/QALY ICUR, The Netherlands); the one by Chao and Chen (13) (ICURs varying from €7715/QALY to €10,826/QALY, Taiwan)‚ and the one by Mandavia et al (14) (incremental net monetary benefit [INMB] of £39,032, United Kingdom). Our goal was to improve the modeling strategy by adding more detail and flexibility in the characterization of HAs nonuse or dropout and to apply it empirically to a cost-utility analysis in the Italian healthcare system, hence contributing to reduce the existing literature gap.

## MATERIALS AND METHODS

A multistate Markov cohort model was developed to evaluate the cost-utility of HAs accompanied by post-purchase service compared to i) no intervention and ii) HA use alone (ie, without post-purchase service) for individuals aged 55 years or older in Italy. The outcomes measured were the ICUR, expressed as the ratio between incremental costs and hearing-related (HR) QALYs, and the INMB, calculated as the difference between incremental benefits, that is, hearing-related QALYs valued at a willingness-to-pay (WTP) threshold, and incremental costs. The WTP threshold used in this study is 16,265 €/QALY (incremental cost per QALY gained), which should reflect the marginal productivity of the Italian healthcare system according to the estimate by Woods et al (15), based on opportunity cost and estimates of the relationship between country gross domestic product per capita and the value of a statistical life. The perspective used is the “restricted” or “limited” societal perspective (Garrison et al [16]), accounting for direct healthcare and nonhealthcare resources consumption and productivity losses. Costs and outcomes were discounted at 3% per year, following economic evaluation recommendations for Italy (17). The model was developed using TreeAge Pro 2019 (18).

### Model Specification

The starting point of the model was set at 55 years and the final point was set at 110 years, consistently with a lifetime horizon, in order to ensure that all relevant differences in costs and outcomes across the interventions under scrutiny are captured. Consistently with the model developed by Chao and Chen (13) and expert opinion, the cycle length is 1 year. Following the modeling strategy proposed by Chao and Chen (13), HL states were defined as normal hearing (<25 decibel [dB]), mild HL (25–44 dB), moderate HL (45–64 dB), and severe-to-profound (from now on “severe”) HL (≥65 dB) in the better hearing ear.

**FIG. 1.**
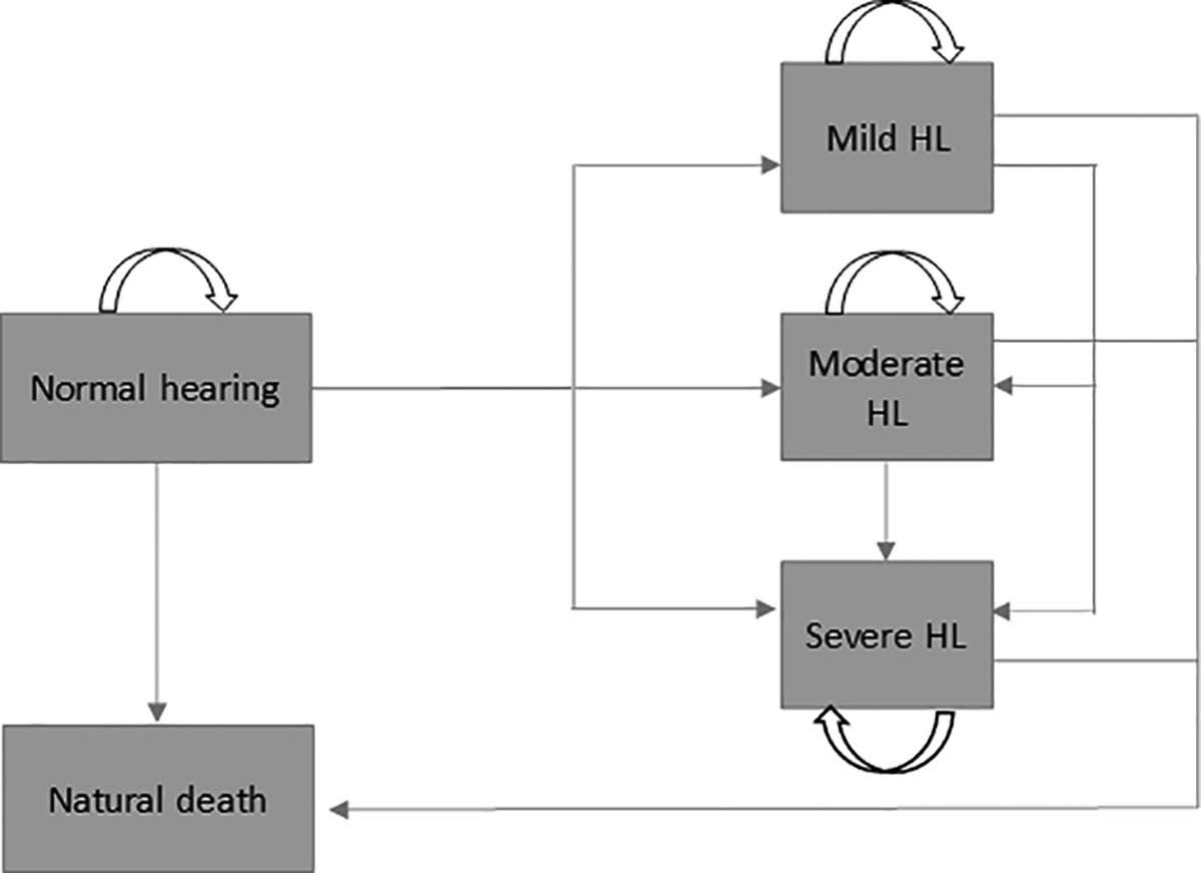
Model diagram, natural history of the disease. HL indicates hearing loss.

In the no intervention arm (ie, the natural history of the disease), normal hearing patients can remain in normal hearing state, progress to mild HL, moderate HL, severe HL or die. If they progress to a HL state, they can remain in such state, progress to a more severe one, or die. The multistate Markov cohort model for the natural history of the disease is graphically shown in Figure 1.

**FIG. 2.**
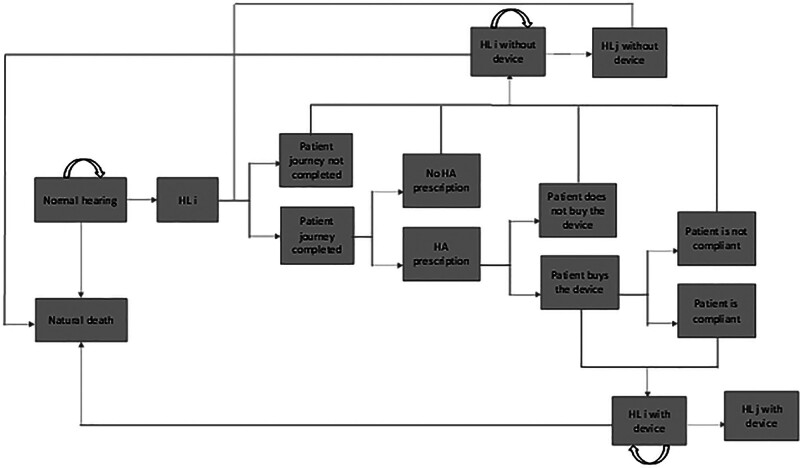
Model diagram, treatment arm. HA indicates hearing aid; HL, hearing loss.

In the treatment arm, when patients experience HL, 4 critical events are considered: i) patients could seek medical help and complete the patient journey up to ear, nose, and throat (ENT) specialist visit or not; ii) if patients complete the patient journey including ENT specialist visit, they could be prescribed a HA or not (perfect prescription appropriateness is assumed); iii) if a HA is prescribed, patients can buy it or not; and iv) if patients buy the HA, they can be compliant (ie, they use the HA as prescribed) or not. If any of these events is not verified, they follow the natural history of the disease without intervention. In case a patient did not complete the patient journey when in HL status i but then progresses to HL status j (j more severe than i), the model allows for the possibility to undergo the patient journey again. If patients are compliant in HA use, it is assumed that they will be compliant also in the subsequent cycles, based on expert opinion. The treatment arm model specification applies to both HA with post-purchase service and HA alone interventions, which differ only for the parameter used to estimate compliance. The multistate Markov cohort model for the treatment arm is graphically shown in Figure 2.

### Hearing Progression Probabilities and Survival

The transition probabilities among different hearing states were based on the results of the systematic literature review and meta-analysis by Chao and Chen (19) and the transition probabilities used in their cost-effectiveness analysis (13). A correction was applied in order for the prevalence predicted by the Markov cohort model to match the real-world HL prevalence estimated in the Rotterdam Study (2011–2015) as in Homans et al (20). Furthermore, transition probabilities were modeled to reflect the fact that the transition across HL states observed in the real world is gradual, meaning that, for example, it is more likely to transition from normal hearing to mild HL than it is to transition from normal hearing to severe HL. The same transition probabilities are applied to both intervention and no intervention arms, as it is assumed that HA use only influences the QoL in a given HL state and not the progression rate across HL states, consistently with other modeling approaches (13) and expert opinion. Gender and age-specific death rates from Italian most recent life tables available published by Istituto Nazionale di Statistica (21) were used to model the transition probability to natural death.

### Patient Journey Completion, HA Prescription, HA Purchase, and Compliance Probabilities

Patient journey completion, HA prescription, HA purchase, and compliance probabilities were estimated using survey data for Italy available from Eurotrak Italy 2018 (10), which provides data from a sample of 15,015 individuals, including both hearing impaired HAs owners and nonowners. According to expert opinion, these probabilities crucially depend on HL severity; for example, patients suffering from severe HL being more likely to seek medical help, receiving HA prescription, purchasing it, and using it if compared to individuals suffering from mild HL. However, stratified data was only available distinguishing between top 50% HL and bottom 50% HL, the former representing more severe patients. As a consequence, estimates from the former subsample were used to characterize severe HL, while estimates from the latter subsample were used to characterize both mild and moderate HL. The rationale is to emphasize the difference in behavior between patients suffering from severe HL compared to milder conditions. As for the probability of being compliant, no stratified data were available; as a consequence, the estimate (94%) was applied irrespectively of HL severity; this approach was deemed realistic also based on empirical data showing that adherence in Italy is the highest among all countries in which Eurotrak surveys are administered (10).

### Quality of Life Improvement After HA Use

Although evidence of statistically significant QoL improvement from HA use is documented in the literature (eg, Lotfi et al [8]), limited data are available in the form of utility weights to be directly applied in cost-utility analyses. Moreover, there exists evidence that the magnitude of QoL estimates varies based on the instrument used to assess them, EuroQol 5 Dimensions scores being on average higher than Health Utility Index 3 scores (eg, Barton et al [22]). As a consequence, 2 different sets of utility weights were used. The first one relies on the estimates of utility weights assigned to grades of HL by Shield (23) (in turn based on Barton et al [22], Davis et al [24], and Swan et al [25]), assuming increments in QoL for HAs use based on Chao and Chen (13); the second one relies on the strategy proposed by Mandavia et al (14), using estimates from Linssen et al (26) and Arndt et al (27).

### Costs

The perspective used was the “restricted” or “limited” societal perspective (Garrison et al [16]), that is, accounting for direct healthcare and nonhealthcare resources consumption and productivity losses.

As for direct healthcare resource consumption, the model accounts for the costs of specialist first and follow-up visits and the cost of the device. In detail, the cost of the first specialist visit is applied if the patient completes the patient journey. Yearly follow-up visits are accounted for if the patient uses the device. The rationale for this choice is that, even though there exists evidence that referral may be delivered by general practitioners, pharmacists, or HAs dispensers, the specialist visit is however necessary to receive a prescription. The model allows to distinguish between the costs of specialists visits delivered through the Italian National Healthcare System (NHS), estimated using Italian published sources (*Nomenclatore dell’assistenza specialistica ambulatoriale* [28]) and the ones delivered privately, estimated based on expert opinion. In the base case, a weighted average of the 2 delivery systems using equal weights was assumed due to lack of reliable estimates on the real-world proportions; this parameter was stressed in the sensitivity analysis. Moreover, it is assumed that if patients comply with using the HA, they need a yearly specialist visit. The cost of the device is applied when patients buy it and, in case they are compliant, it is assumed to be replaced each 5 years. Separate estimations were performed considering the costs for specialist visits and the device supplied i) exclusively in the private market, ii) exclusively through the Italian NHS, and iii) exclusively through the Italian NHS assuming that the tariff reimbursed by the Italian NHS coincides with the price applied by the private suppliers. The price for the private market includes the post-purchase service, as it is conceived as part of the bundle of services connected to the device provision. No costs for battery replacement were considered because, according to expert opinion, the patients’ preferences have shifted towards devices that can be recharged at home. The natural obsolescence of the included battery is captured by the replacement of the device every 5 years.

Given the paucity of data on the costs and comparative risks of social isolation, depression, and dementia, it was assumed that these dimensions are captured by the utility weights applied to the HL states. Data on age-specific annual incidence of unintentional falls in older people leading to accident and emergency attendances was retrieved from Scuffham et al (29), Girard et al (30), and Mahmoudi et al (31). The cost for injurious falls leading to hospitalization was operationalized using the tariff associated to the diagnosis-related group (DRG) for femur fracture (32). As for direct nonhealthcare costs, transportation costs were applied to each specialist visit, and, following Chao and Chen (13), were arbitrarily set at €5.84.

As for productivity losses, it is recognized in the literature that HL can negatively impact on employment and career opportunity. The estimate based on real-world data of $48 per 7 months as excess occupational health expenditure by Nachtegaal et al (33) was applied to unaided moderate and severe HL states up to 64 years of age; as a proxy for the mitigating effect of HA use, a reduction equal to the percentage difference between unemployment rates in unaided and aided hearing impaired individuals estimated by Kochkin (34) was used. Finally, following Chao and Chen (13), the loss of productivity for the days in which specialist visits take place was estimated using Italian daily gross national product per capita (35). All costs are reported in 2021 Euros. The complete list of model inputs is reported in Table 1.

**TABLE 1.** Model inputs

Parameter	Input value	Source
Clinical pathway probabilities		
Patient journey completion, mild HL	0.2700	Eurotrak Italy 2018 (10)
Patient journey completion, moderate HL	0.2700	Eurotrak Italy 2018 (10)
Patient journey completion, severe HL	0.5400	Eurotrak Italy 2018 (10)
Probability of receiving HA prescription, mild HL	0.7037	Computations on Eurotrak Italy 2018 (10)
Probability of receiving HA prescription, moderate HL	0.7037	Computations on Eurotrak Italy 2018 (10)
Probability of receiving HA prescription, severe HL	0.9259	Computations on Eurotrak Italy 2018 (10)
Probability of purchasing the device, mild HL	0.8421	Computations on Eurotrak Italy 2018 (10)
Probability of purchasing the device, moderate HL	0.8421	Computations on Eurotrak Italy 2018 (10)
Probability of purchasing the device, severe HL	0.9400	Computations on Eurotrak Italy 2018 (10)
Probability of using the device, mild HL	0.9400	Eurotrak Italy 2018 (10)
Probability of using the device, moderate HL	0.9400	Eurotrak Italy 2018 (10)
Probability of using the device, severe HL	0.9400	Eurotrak Italy 2018 (10)
Expected decrement in compliance in the absence of post-purchase service	–0.3600	Boas et al 2001 (11)
Utility weights (base case)		
Mild HL, unaided	0.8000	Shield 2018 (23)
Moderate HL, unaided	0.6500	Computations on Shield 2018 (23)
Severe HL, unaided	0.4500	Computations on Shield 2018 (23)
Mild HL, aided	0.9300	Computations on Shield 2018 (23), Chao and Chen 2008 (13)
Moderate HL, aided	0.9300	Computations on Shield 2018 (23), Chao and Chen 2008 (13)
Severe HL, aided	0.6900	Computations on Shield 2018 (23), Chao and Chen 2008 (13)
Utility weights (alternative set)		
Mild HL, unaided	0.8100	Mandavia et al 2020 (14), Linssen et al 2013 (26)
Moderate HL, unaided	0.7700	Mandavia et al 2020 (14), Linssen et al 2013 (26)
Severe HL, unaided	0.6200	Mandavia et al 2020 (14), Linssen et al 2013 (26), Arndt et al 2011 (27)
Mild HL, aided	0.9000	Mandavia et al 2020 (14), Linssen et al 2013 (26)
Moderate HL, aided	0.8600	Mandavia et al 2020 (14), Linssen et al 2013 (26)
Severe HL, aided	0.7100	Mandavia et al 2020 (14), Linssen et al 2013 (26)
Other parameters		
Frequency of specialist follow-up visits (y)	1	Expert opinion
Frequency of device replacement (y)	5	CEIS 2020 (36)
Probability of unemployment with unaided moderate HL	0.1070	Kochkin 2010 (34)
Probability of unemployment with unaided severe HL	0.1560	Kochkin 2010 (34)
Probability of unemployment with aided moderate HL	0.0540	Kochkin 2010 (34)
Probability of unemployment with aided severe HL	0.0820	Kochkin 2010 (34)
Discount factor	0.03	Capri et al 2001 (17)
Willingness-to-pay threshold (€/QALY)	16,265	Woods et al 2016 (15)
*Mercato sociale* proportion	0.2000	CEIS 2020 (30)
*Mercato riconducibile* proportion	0.2600	CEIS 2020 (30)
Proportion of public specialist visits	0.5000	Assumption
Injurious falls leading to hospitalization annual incidence, 60–64 y	0.0035	Scuffham et al 2003 (29)
Injurious falls leading to hospitalization annual incidence, 65–69 y	0.0052	Scuffham et al 2003 (29)
Injurious falls leading to hospitalization annual incidence, 70–74 y	0.0092	Scuffham et al 2003 (29)
Injurious falls annual incidence, ≥75 y	0.0369	Scuffham et al 2003 (29)
Injurious falls leading to hospitalization in the elderly unaided hearing impaired, odds ratio	1.9700	Girard et al 2014 (30)
Injurious falls leading to hospitalization in the elderly aided hearing impaired, hazard ratio	0.8700	Mahmoudi et al 2019 (31)
Costs		
First specialist visit, public (€)	29.00	Nomenclatore dell’assistenza specialistica ambulatoriale 89.7B.9 (28)
First specialist visit, private (€)	125.00	Expert opinion
Follow-up specialist visit, public (€)	17.00	Nomenclatore dell’assistenza specialistica ambulatoriale 89.7B.9 (28)
Follow-up specialist visit private (€)	75.00	Expert opinion
Device (private market price, “standard” category) (€)	1990.90	Expert opinion
Device (public reimbursement tariff)	672.50	Nomenclatore tariffario D.M. 332/1999 (37)
Femur fracture DRG tariff (€)	6099.00	Tariffe delle prestazioni per acuti per tipo di ricovero (32)
Transportation costs (€)	5.84	Chao and Chen 2008 (13)
Annual excess occupational health expenditure for the hearing impaired (€)	82.87	Computations on Nachtegaal et al 2010 (33)
Daily gross national product per capita (€)	71.73	ISTAT 2021 (35)

DRG indicates diagnosis-related group; HA, hearing aid; HL, hearing loss; LY, life years; NMB, net monetary benefit; pps, post-purchase service; QALY, quality-adjusted life year.

### Sensitivity and Scenario Analysis

Sensitivity analysis was performed in order to assess the robustness of the model to variations of each key parameter. In deterministic sensitivity analysis (DSA) parameters were varied by assumed plausible ranges and each variable was tested at the lower and upper limit of its selected interval. Results were reported in a Tornado diagram on INMB. Probabilistic sensitivity analysis (PSA) was performed using a Monte-Carlo simulation with 1‚000 iterations. Results were reported in a cost-effectiveness acceptability curve (CEAC), representing the probability of an intervention being cost-effective over a range of different WTP thresholds, and in a cost-effectiveness plane.

Extensive details on methods, parameters and range of variation are reported in the Supplemental Materials (http://links.lww.com/ONO/A5).

Scenario analyses were conducted in order to test how the outcomes of interest would change under different utility weights sets and device pricing, as stated above. Moreover, ICUR and INMB were computed and compared in the following scenarios: i) current mix in terms of dropout (ie, patients not completing the patient journey or not purchasing the device) and compliance, ii) no dropout but partial compliance, and iii) no dropout and full compliance.

## RESULTS

Base-case results are reported in Table 2.

**TABLE 2.** Base-case results

Gender: male			Gender: female		
Outcome	HA + pps	HA	Outcome	HA + pps	HA
Mean costs (€)	2‚339	2‚243	Mean costs (€)	2‚764	2‚664
Mean LYs	18.4096	18.4096	Mean LYs	20.0094	20.0094
Mean QALYs	17.4070	17.3445	Mean QALYs	18.9073	18.8416
NMB	280,787	279,866	NMB	304,764	303,795
Compared with HA w/out post-purchase service			Compared with HA w/out post-purchase service		
Incremental costs (€)	96		Incremental costs (€)	100	
Incremental QALYs	0.0625		Incremental QALYs	0.0657	
ICUR (€/QALYs)	1‚531		ICUR (€/QALYs)	1‚521	
INMB	921		INMB	969	
Outcome	HA + pps	No treat	Outcome	HA + pps	No treat
Mean costs (€)	2‚339	1‚909	Mean costs (€)	2‚764	2‚288
Mean LYs	18.4096	18.4096	Mean LYs	20.0094	20.0094
Mean QALYs	17.4070	17.2284	Mean QALYs	18.9073	18.7132
NMB	280,787	278,311	NMB	304,764	302,082
Compared with no treatment			Compared with no treatment		
Incremental costs (€)	429		Incremental costs (€)	476	
Incremental QALYs	0.1786		Incremental QALYs	0.1941	
ICUR (€/QALYs)	2‚404		ICUR (€/QALYs)	2‚450	
INMB	2‚476		INMB	2‚682	
Outcome	HA	No treat	Outcome	HA	No treat
Mean costs (€)	2‚243	1‚909	Mean costs (€)	2‚664	2‚288
Mean LYs	18.4096	18.4096	Mean LYs	20.0094	20.0094
Mean QALYs	17.3445	17.2284	Mean QALYs	18.8416	18.7132
NMB	279,866	278,311	NMB	303,795	302,082
Compared with no treatment			Compared with no treatment		
Incremental costs (€)	334		Incremental costs (€)	376	
Incremental QALYs	0.1161		Incremental QALYs	0.1284	
ICUR (€/QALYs)	2‚875		ICUR (€/QALYs)	2‚925	
INMB	1‚555		INMB	1‚713	

HA indicates hearing aid; ICUR, incremental cost-utility ratio; INMB, incremental net monetary benefit; LY, life years; NMB, net monetary benefit; pps, post-purchase service; QALY, quality-adjusted life year.

In the base case, with the current dropout mix, HAs use (including post-purchase service) compared to no treatment results in an incremental cost of €429 and €476 in the male and female cohort, respectively, and incremental hearing-related (HR) QALY gain of 0.18 and 0.19, respectively, leading to estimated ICURs of €2‚404/QALY and €2‚450/QALY, respectively, and INMB of €2‚476 and €2‚682, respectively. In the no dropout scenario, both incremental costs and incremental HR QALYs gained increase (€1‚659–€1‚763 and 0.77–0.79); however, it is worth noticing that this scenario is characterized by a relevant HR QALYs gain (+428% for males and +416% for females) with a relatively modest increase in ICURs (€2‚148/QALY–€2‚245/QALY)—still well below the WTP threshold—and a larger increase in INMB (€10,901–€11,015). This trend is reinforced when full compliance is added to absence of dropout. These results are robust to the alternative utility weights set (ie, the approach by Mandavia et al [14]), where incremental HR QALYs gained are smaller, yielding to larger ICURs (still well below the WTP threshold) and relatively smaller INMB. By considering only private market costs, incremental costs increase up to €2‚397–€2‚591 but the treatment still maintains a cost-effective profile (the largest ICURs estimated are €5‚750/QALY–€5‚546/QALY). The complete set of results of the scenario analysis for the different mixes in terms of costs and dropout/compliance rates are reported in the Supplemental Materials (http://links.lww.com/ONO/A5).

**FIG. 3.**
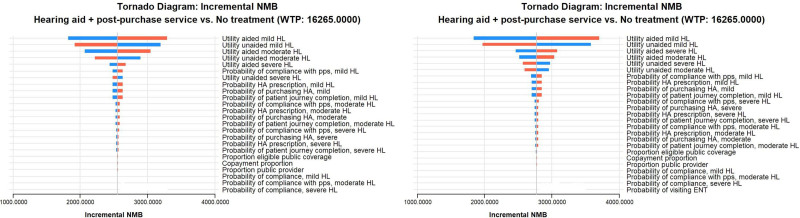
Tornado diagram. Left: male cohort; right: female cohort. ENT indicates ear, nose, and throat; HL, hearing loss; INMB, incremental net monetary benefit; pps‚ post-purchase service.

DSA shows that INMB estimates are robust to parameter variations, with utility weights both in aided and unaided HL states being the most influential parameters both for the male and female cohort. INMB tornado diagrams are reported in Figure 3.

**FIG. 4.**
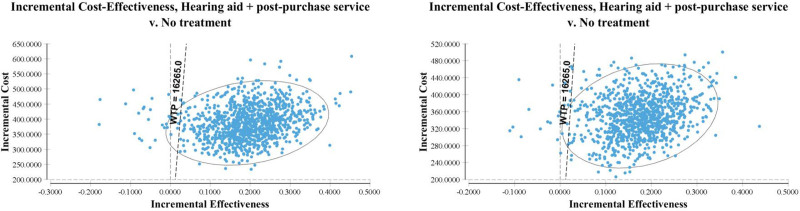
Cost-effectiveness plane. Left: male cohort; right: female cohort. WTP indicates willingness to pay.

**FIG. 5.**
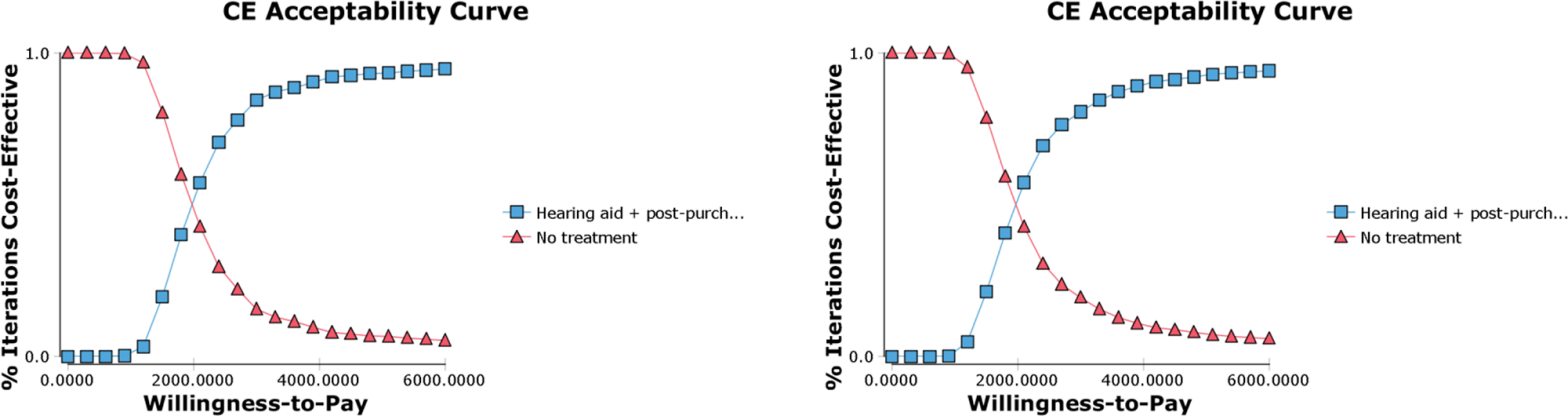
Acceptability curve. Left: male cohort; right: female cohort. CE indicates cost effectiveness.

PSA results confirm that the cost-effectiveness profile is robust to parameters variations, with a probability of being cost-effective at a €16,625/QALY WTP threshold of 97.8% and 97.3% for the male and female cohorts, respectively, in the base case; cost-effectiveness planes and CEACs are reported in Figure 4 and Figure 5.

## DISCUSSION

Our model suggests that the intervention is cost-effective when contrasted to a €16,625/QALY WTP threshold and results are robust to deterministic and probabilistic parameter variations and to different cost and compliance assumptions explored in scenario analyses. Our results are consistent with those featured in the peer-reviewed literature on the subject (eg, Chao and Chen [13], Joore et al [12], Boas et al [11]). If compared to the work by Chao and Chen (13), which is the closest to ours in scope and modeling structure, incremental costs, HR QALY gains and ICURs are smaller in the base case; this feature may be due to the different cost structure reflective of different healthcare systems and mostly to the different model structure. Indeed, instead of limiting “satisfaction with HA use” to be the only driver for HA actual use, we distinguish 4 crucial stages that are potential sources of dropout from HA use. It is worth noticing, indeed, that when we move from the current dropout mix to the assumption of no dropout, incremental HR QALYs gained and ICURs get closer to the ones estimated by Chao and Chen (13). We believe our model structure represents an advancement in the literature because it allows larger flexibility in exploring the drivers of HAs nonuse, for example, by testing the effects of potential interventions affecting the probability of patient journey completion, HA prescription, HA purchase, and “*ex-post*” compliance that can be implemented by both public and private healthcare providers.

The model presents limitations that need to be acknowledged. The greatest limitation is related to the scarcity of reliable secondary data suitable to populate the model. This limitation affects primarily the utility weights attached to the different health states, which represent the major source of variability as confirmed by the sensitivity analysis. Moreover, prevalence data and data on HAs use are rarely stratified by disease severity; on a similar line, if‚ on the one hand, the detrimental impact of HL on social isolation, depression, dementia, employability, and productivity is documented‚ on the other hand, very little evidence exists on how HAs use mitigates these effects. Furthermore, although the average natural obsolescence of the battery included in the device is captured by the replacement of the device each 5 years, it can be argued that batteries may need to be replaced more frequently; however, due to lack of referenced real-world estimates, this cost item was not fully accounted for in the analysis. Finally, it can be argued that specialist visits provided by the NHS, although more affordable, entail on average longer waiting times compared to private providers and the model fails to capture this aspect; to mitigate the bias, a scenario using private providers' prices only was estimated.

As a direction for future research, generating patient preferences and QoL estimates should be considered as a priority, given the fact that they represent the most impactful parameter in the sensitivity analysis.

## CONCLUSIONS

Our multistate Markov cohort model indicates that HAs use for individuals aged at least 55 years in the context of the Italian healthcare system is a cost-effective strategy to address HL, reinforcing the importance of policies that facilitate early uptake. Our model may represent an advancement in the literature by allowing the flexibility to analyze the impact of different factors on HAs nonuse, which can inform pricing and reimbursement decisions and resource allocation. Future research in the field should prioritize the generation of reliable effectiveness and comparative risk data to improve the ability to assess the cost-utility of HAs.

## FUNDING SOURCES

Funding received for this work from any of the following organizations: National Institutes of Health (NIH), Wellcome Trust, Howard Hughes Medical Institute (HHMI), and other(s): CERGAS SDA Bocconi obtained an unconditional funding by Amplifon Italy for this research.

## CONFLICT OF INTEREST

G.F. received a personal honorarium by Amplifon Italy in 2021 to present the results of this study during the 107th Symposium of Società Italiana di Otorinolaringoiatria (SIO). A.A. is a member of Amplifon Italy’s CRS board. The remaining author discloses no conflicts of interest.

## DATA AVAILABILITY STATEMENT

The datasets generated during and/or analyzed during the current study are publicly available.

## Supplementary Material


