# Supplementary material for: The Value of Hearing Aids for the Italian NHS: A Cost-utility Analysis
Source: Otol Neurotol Open. 2022 Oct 26;2(4):e018. doi: 10.1097/ONO.0000000000000018 (PMC10950133; doi:10.1097/ONO.0000000000000018)
Supplement: Supplementary file 1 [file ono-2-e018-s001.pdf]

Supplemental materials

Index

Model specification ... 2

Hearing loss progression and survival ... 3

Review of published economic evaluations (more details) ... 15

Patient journey completion, hearing aids prescription, hearing aids purchase and compliance probabilities (more details) ... 15

Quality of life improvement after HA use (more details) ... 16

Costs (more details) ... 17

Sensitivity analysis ... 18

Scenario analysis results ... 20

CHEERS checklist ... 57

References ... 60

Model specification

Figure 1 Full-fledged TreeAge model structure, hearing aid (plus post-purchase service) arm

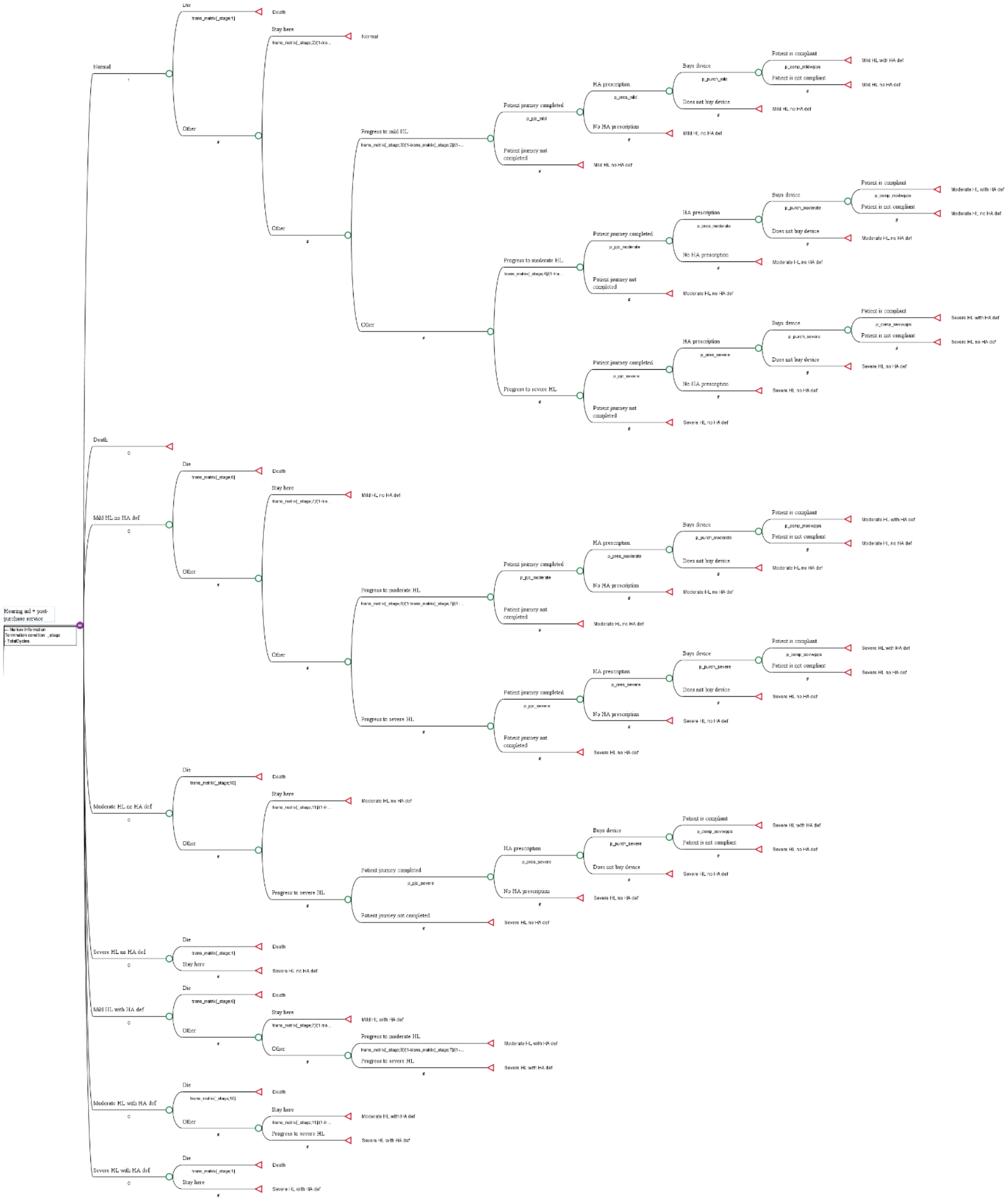

Figure 2 Full-fledged TreeAge model structure, no treatment arm

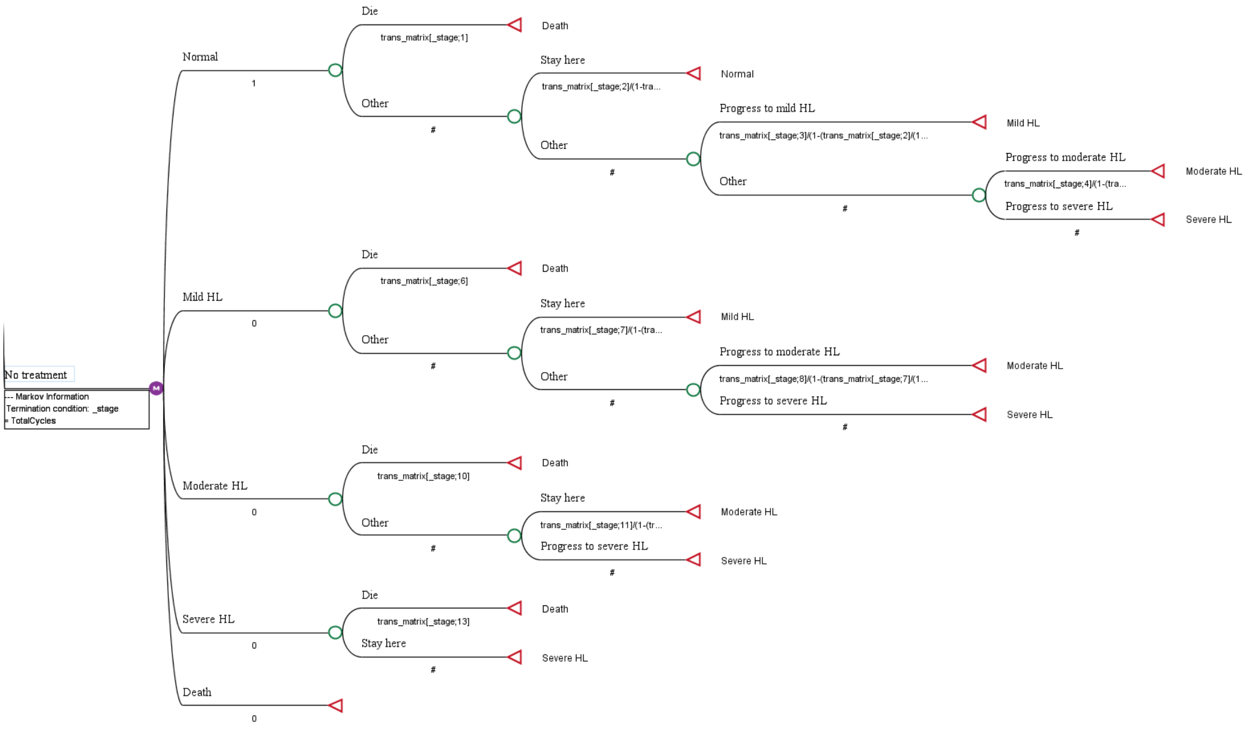

Hearing loss progression and survival

Table 1 Markov cohort results, male cohort

Age	Normal hearing	Mild hearing loss	Moderate hearing loss	Severe hearing loss	Dead
55	100	0	0	0	0
56	96	4	0	0	0
57	92	7	0	0	1
58	88	10	1	0	1
59	84	12	1	1	2
60	80	15	2	1	2
61	76	17	3	1	3
62	75	17	4	1	4
63	74	17	4	1	4
64	73	17	4	1	5
65	71	18	4	1	6
66	70	18	5	1	7
67	68	18	5	1	8

68	67	18	5	1	9
69	65	18	5	1	10
70	64	18	6	1	12
71	62	18	6	1	13
72	59	18	6	1	15
73	57	18	7	1	16
74	55	18	7	1	18
75	53	18	8	1	20
76	51	18	8	1	22
77	48	18	8	1	25
78	46	17	8	1	27
79	43	17	9	1	30
80	41	16	9	1	33
81	38	16	9	1	36
82	35	15	9	1	40
83	33	14	9	1	43
84	30	13	8	1	47
85	27	12	8	1	51
86	24	11	8	1	56
87	21	10	7	1	60
88	18	9	7	1	65
89	15	8	6	1	69
90	13	7	6	1	74
91	11	5	5	1	78
92	8	4	4	1	82
93	7	4	3	1	86
94	5	3	3	1	89
95	4	2	2	1	92
96	3	1	2	0	94
97	2	1	1	0	96
98	1	1	1	0	97

99	1	0	1	0	98
100	0	0	0	0	99
101	0	0	0	0	99
102	0	0	0	0	99
103	0	0	0	0	100
104	0	0	0	0	100
105	0	0	0	0	100
106	0	0	0	0	100
107	0	0	0	0	100
108	0	0	0	0	100
109	0	0	0	0	100
110	0	0	0	0	100

Table 2 Predicted hearing loss prevalence compared to real world hearing loss prevalence (the Rotterdam Study, 2011-20151), male cohort

Age	Predicted hearing loss prevalence	Real world hearing loss prevalence (the Rotterdam Study, 2011-2015[1])
60	0.1805	0.1990
65	0.2429	0.2380
70	0.2794	0.2800
75	0.3381	0.3305
80	0.3928	0.3835
85	0.4444	0.4475

Figure 3 Markov probability analysis, male cohort. Top left: hearing aid plus post-purchase service; top right: hearing aid alone; bottom: no treatment

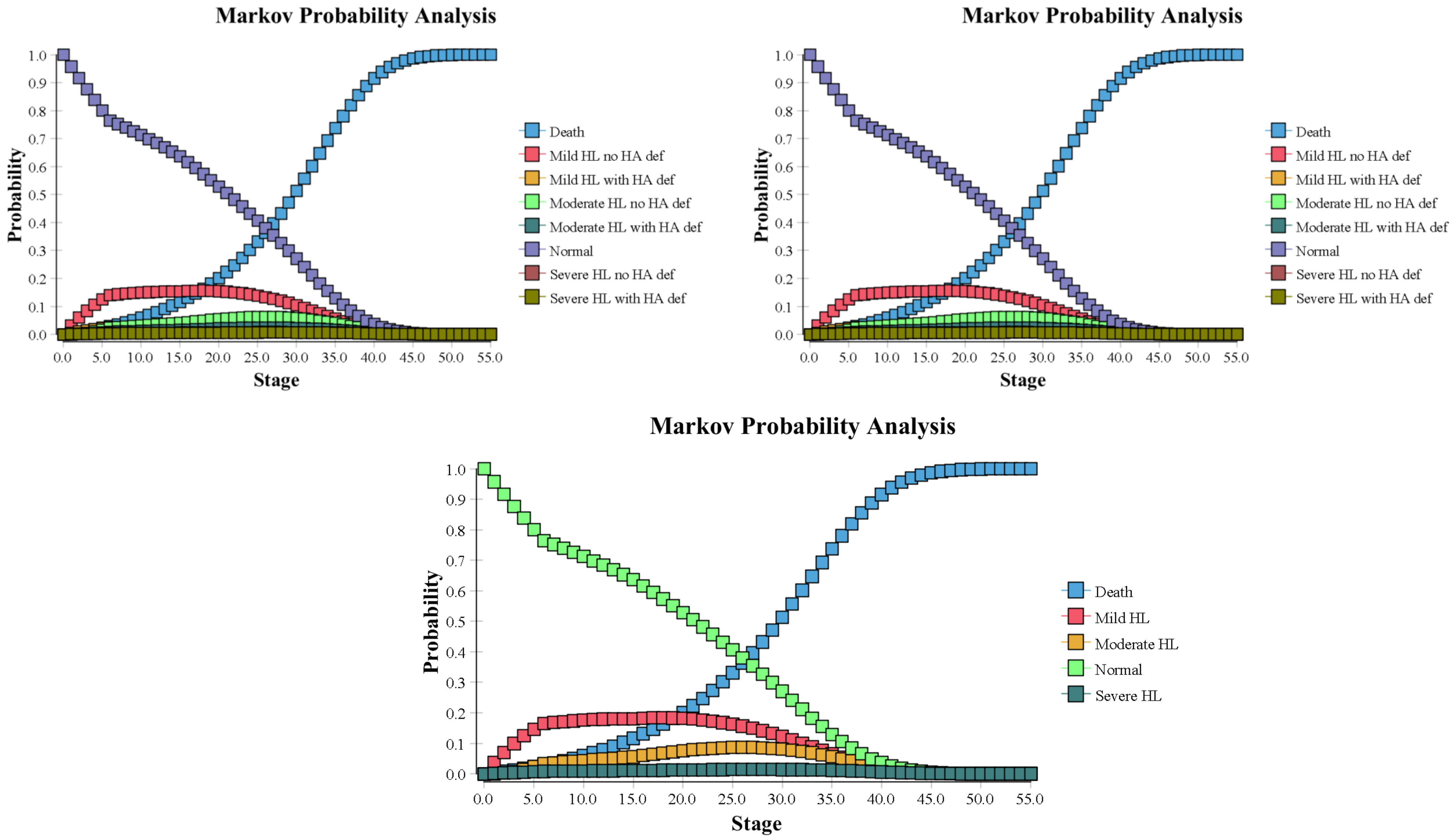

Table 3. Transition matrix, male cohort

Age	NH --> D	NH --> NH	NH --> MIHL	NH --> MOHL	NH --> SHL	MIHL --> D	MIHL --> MIHL	MIHL --> MOHL	MIHL --> SHL	MOHL --> D	MOHL --> MOHL	MOHL --> SHL	SHL --> D	SHL --> SHL
55	0.00382	0.95734	0.03746	0.00137	0.00001	0.00382	0.92682	0.06934	0.00002	0.00382	0.99575	0.00043	0.00382	0.99618
56	0.00413	0.95702	0.03746	0.00137	0.00001	0.00413	0.92650	0.06934	0.00002	0.00413	0.99544	0.00043	0.00413	0.99587
57	0.00460	0.95656	0.03746	0.00137	0.00001	0.00460	0.92603	0.06935	0.00002	0.00460	0.99497	0.00043	0.00460	0.99540
58	0.00508	0.95607	0.03747	0.00137	0.00001	0.00508	0.92555	0.06935	0.00002	0.00508	0.99449	0.00043	0.00508	0.99492
59	0.00555	0.95560	0.03747	0.00137	0.00001	0.00555	0.92507	0.06935	0.00002	0.00555	0.99402	0.00043	0.00555	0.99445
60	0.00619	0.95496	0.03747	0.00137	0.00001	0.00619	0.92443	0.06936	0.00002	0.00619	0.99338	0.00043	0.00619	0.99381
61	0.00680	0.98349	0.00937	0.00034	0.00000	0.00680	0.97585	0.01734	0.00001	0.00680	0.99309	0.00011	0.00680	0.99320
62	0.00756	0.98273	0.00937	0.00034	0.00000	0.00756	0.97510	0.01734	0.00001	0.00756	0.99233	0.00011	0.00756	0.99244
63	0.00821	0.98208	0.00937	0.00034	0.00000	0.00821	0.97445	0.01734	0.00001	0.00821	0.99168	0.00011	0.00821	0.99179
64	0.00905	0.98124	0.00937	0.00034	0.00000	0.00905	0.97361	0.01734	0.00001	0.00905	0.99085	0.00011	0.00905	0.99095
65	0.00994	0.98035	0.00937	0.00034	0.00000	0.00994	0.97272	0.01734	0.00001	0.00994	0.98996	0.00011	0.00994	0.99006
66	0.01113	0.97915	0.00937	0.00034	0.00000	0.01113	0.97152	0.01734	0.00001	0.01113	0.98876	0.00011	0.01113	0.98887
67	0.01236	0.97793	0.00937	0.00034	0.00000	0.01236	0.97030	0.01734	0.00001	0.01236	0.98754	0.00011	0.01236	0.98764
68	0.01383	0.97646	0.00937	0.00034	0.00000	0.01383	0.96883	0.01734	0.00001	0.01383	0.98606	0.00011	0.01383	0.98617
69	0.01506	0.97523	0.00937	0.00034	0.00000	0.01506	0.96760	0.01734	0.00001	0.01506	0.98483	0.00011	0.01506	0.98494
70	0.01682	0.96667	0.01592	0.00058	0.00000	0.01682	0.95370	0.02948	0.00001	0.01682	0.98300	0.00018	0.01682	0.98318
71	0.01777	0.96572	0.01592	0.00058	0.00000	0.01777	0.95274	0.02948	0.00001	0.01777	0.98204	0.00018	0.01777	0.98223
72	0.01937	0.96412	0.01592	0.00058	0.00000	0.01937	0.95114	0.02948	0.00001	0.01937	0.98045	0.00018	0.01937	0.98063
73	0.02152	0.96197	0.01592	0.00058	0.00000	0.02152	0.94899	0.02948	0.00001	0.02152	0.97829	0.00018	0.02152	0.97848
74	0.02441	0.95908	0.01592	0.00058	0.00000	0.02441	0.94610	0.02948	0.00001	0.02441	0.97541	0.00018	0.02441	0.97559
75	0.02692	0.95657	0.01592	0.00058	0.00000	0.02692	0.94359	0.02948	0.00001	0.02692	0.97289	0.00018	0.02692	0.97308
76	0.03111	0.95238	0.01592	0.00058	0.00000	0.03111	0.93941	0.02948	0.00001	0.03111	0.96871	0.00018	0.03111	0.96889
77	0.03484	0.94865	0.01592	0.00058	0.00000	0.03484	0.93568	0.02948	0.00001	0.03484	0.96498	0.00018	0.03484	0.96516
78	0.03869	0.94480	0.01592	0.00058	0.00000	0.03869	0.93182	0.02948	0.00001	0.03869	0.96113	0.00018	0.03869	0.96131
79	0.04301	0.94048	0.01592	0.00058	0.00000	0.04301	0.92750	0.02948	0.00001	0.04301	0.95681	0.00018	0.04301	0.95699
80	0.04694	0.93655	0.01592	0.00058	0.00000	0.04694	0.92357	0.02948	0.00001	0.04694	0.95287	0.00018	0.04694	0.95306

81	0.05209	0.93139	0.01592	0.00058	0.00000	0.05209	0.91842	0.02948	0.00001	0.05209	0.94772	0.00018	0.05209	0.94791
82	0.05962	0.92386	0.01592	0.00058	0.00000	0.05962	0.91089	0.02948	0.00001	0.05962	0.94019	0.00018	0.05962	0.94038
83	0.06884	0.91465	0.01592	0.00058	0.00000	0.06884	0.90168	0.02948	0.00001	0.06884	0.93098	0.00018	0.06884	0.93116
84	0.07860	0.90489	0.01592	0.00058	0.00000	0.07860	0.89192	0.02948	0.00001	0.07860	0.92122	0.00018	0.07860	0.92140
85	0.08922	0.88931	0.02070	0.00076	0.00001	0.08922	0.87245	0.03832	0.00001	0.08922	0.91054	0.00024	0.08922	0.91078
86	0.10147	0.87706	0.02070	0.00076	0.00001	0.10147	0.86020	0.03832	0.00001	0.10147	0.89829	0.00024	0.10147	0.89853
87	0.11529	0.86324	0.02070	0.00076	0.00001	0.11529	0.84638	0.03832	0.00001	0.11529	0.88447	0.00024	0.11529	0.88471
88	0.12921	0.84933	0.02070	0.00076	0.00001	0.12921	0.83246	0.03832	0.00001	0.12921	0.87056	0.00024	0.12921	0.87079
89	0.14579	0.83275	0.02070	0.00076	0.00001	0.14579	0.81588	0.03832	0.00001	0.14579	0.85398	0.00024	0.14579	0.85421
90	0.16168	0.81686	0.02070	0.00076	0.00001	0.16168	0.79999	0.03832	0.00001	0.16168	0.83808	0.00024	0.16168	0.83832
91	0.18044	0.79810	0.02070	0.00076	0.00001	0.18044	0.78123	0.03832	0.00001	0.18044	0.81932	0.00024	0.18044	0.81956
92	0.20114	0.77740	0.02070	0.00076	0.00001	0.20114	0.76053	0.03832	0.00001	0.20114	0.79862	0.00024	0.20114	0.79886
93	0.22392	0.75462	0.02070	0.00076	0.00001	0.22392	0.73775	0.03832	0.00001	0.22392	0.77585	0.00024	0.22392	0.77608
94	0.24481	0.73372	0.02070	0.00076	0.00001	0.24481	0.71686	0.03832	0.00001	0.24481	0.75495	0.00024	0.24481	0.75519
95	0.27018	0.70406	0.02484	0.00091	0.00001	0.27018	0.68382	0.04598	0.00001	0.27018	0.72953	0.00029	0.27018	0.72982
96	0.29201	0.68223	0.02484	0.00091	0.00001	0.29201	0.66199	0.04598	0.00001	0.29201	0.70771	0.00029	0.29201	0.70799
97	0.30517	0.66907	0.02484	0.00091	0.00001	0.30517	0.64883	0.04598	0.00001	0.30517	0.69454	0.00029	0.30517	0.69483
98	0.31690	0.65734	0.02484	0.00091	0.00001	0.31690	0.63710	0.04598	0.00001	0.31690	0.68282	0.00029	0.31690	0.68310
99	0.33417	0.64007	0.02484	0.00091	0.00001	0.33417	0.61983	0.04598	0.00001	0.33417	0.66555	0.00029	0.33417	0.66583
100	0.36601	0.60824	0.02484	0.00091	0.00001	0.36601	0.58800	0.04598	0.00001	0.36601	0.63371	0.00029	0.36601	0.63399
101	0.41581	0.55844	0.02484	0.00091	0.00001	0.41581	0.53820	0.04598	0.00001	0.41581	0.58391	0.00029	0.41581	0.58419
102	0.44869	0.52555	0.02484	0.00091	0.00001	0.44869	0.50531	0.04598	0.00001	0.44869	0.55103	0.00029	0.44869	0.55131
103	0.48215	0.49209	0.02484	0.00091	0.00001	0.48215	0.47185	0.04598	0.00001	0.48215	0.51756	0.00029	0.48215	0.51785
104	0.51591	0.45833	0.02484	0.00091	0.00001	0.51591	0.43809	0.04598	0.00001	0.51591	0.48380	0.00029	0.51591	0.48409
105	0.54967	0.42457	0.02484	0.00091	0.00001	0.54967	0.40433	0.04598	0.00001	0.54967	0.45004	0.00029	0.54967	0.45033
106	0.58314	0.39110	0.02484	0.00091	0.00001	0.58314	0.37086	0.04598	0.00001	0.58314	0.41658	0.00029	0.58314	0.41686
107	0.61601	0.35823	0.02484	0.00091	0.00001	0.61601	0.33799	0.04598	0.00001	0.61601	0.38370	0.00029	0.61601	0.38399
108	0.64803	0.32621	0.02484	0.00091	0.00001	0.64803	0.30597	0.04598	0.00001	0.64803	0.35169	0.00029	0.64803	0.35197
109	0.67893	0.29531	0.02484	0.00091	0.00001	0.67893	0.27507	0.04598	0.00001	0.67893	0.32078	0.00029	0.67893	0.32107

Key. D = death, MIHL = mild hearing loss, MOHL = moderate hearing loss, NH = normal hearing, SHL = severe hearing loss

Table 4 Markov cohort results, female cohort

Age	Normal hearing	Mild hearing loss	Moderate hearing loss	Severe hearing loss	Dead
55	100	0	0	0	0
56	95	4	0	0	0
57	91	8	0	0	0
58	87	11	1	0	1
59	84	14	1	0	1
60	81	16	1	0	1
61	77	18	2	1	2
62	76	19	2	1	2
63	75	19	2	1	2
64	74	19	2	1	3
65	73	20	2	1	3
66	72	20	3	1	4
67	71	20	3	1	5
68	70	21	3	1	5
69	69	21	3	1	6
70	68	21	3	1	7
71	66	22	3	2	7
72	64	22	3	2	8
73	63	22	3	2	9
74	61	23	4	2	10
75	59	23	4	3	12
76	57	23	4	3	13
77	56	23	4	3	15
78	54	23	4	3	16
79	52	23	4	3	18
80	49	22	4	4	20
81	47	22	4	4	23
82	45	21	4	4	25
83	42	21	4	4	28

84	40	20	4	4	32
85	37	19	4	4	35
86	34	18	4	4	39
87	31	17	4	5	44
88	28	16	4	5	48
89	24	14	3	4	53
90	21	13	3	4	59
91	18	11	3	4	64
92	15	10	2	4	69
93	13	8	2	3	74
94	10	7	2	3	78
95	8	5	1	3	83
96	6	4	1	2	87
97	4	3	1	2	90
98	3	2	1	1	92
99	2	2	0	1	95
100	1	1	0	1	96
101	1	1	0	1	97
102	1	0	0	0	98
103	0	0	0	0	99
104	0	0	0	0	99
105	0	0	0	0	100
106	0	0	0	0	100
107	0	0	0	0	100
108	0	0	0	0	100
109	0	0	0	0	100
110	0	0	0	0	100

Table 5 Predicted hearing loss prevalence compared to real world hearing loss prevalence (the Rotterdam Study, 2011-20151), male cohort

Age	Predicted hearing loss prevalence	Real world hearing loss prevalence (the Rotterdam Study, 2011-2015[1])
60	0.1828	0.1793
65	0.2402	0.2201
70	0.2739	0.2610
75	0.3282	0.3124
80	0.3789	0.3657
85	0.4267	0.4225

Figure 4 Markov probability analysis, female cohort. Top left: hearing aid plus post-purchase service; top right: hearing aid alone; bottom: no treatment

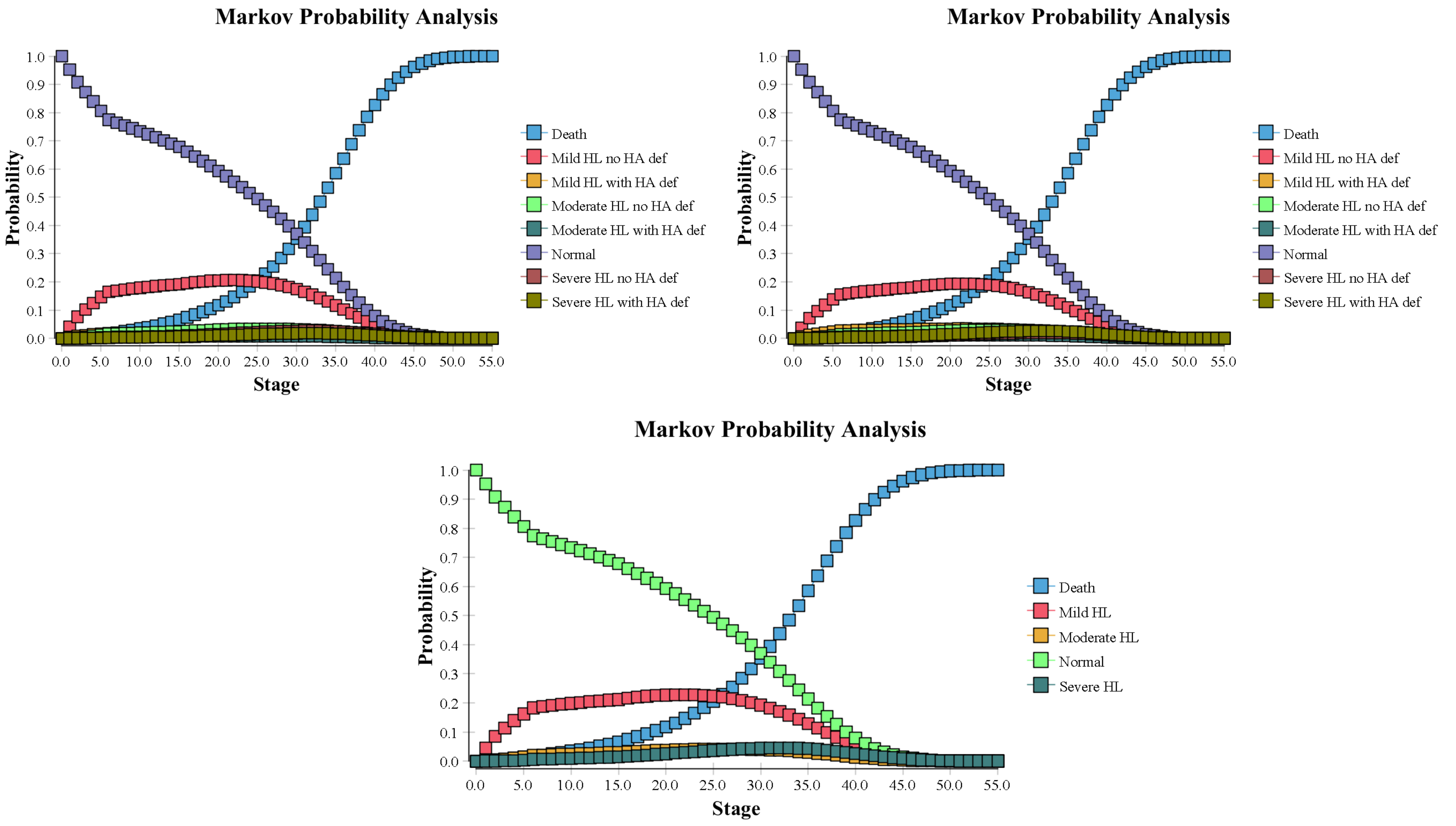

Table 6 Transition matrix, female cohort

Age	NH --> D	NH --> NH	NH --> MIHL	NH --> MOHL	NH --> SHL	MIHL --> D	MIHL --> MIHL	MIHL --> MOHL	MIHL --> SHL	MOHL --> D	MOHL --> MOHL	MOHL --> SHL	SHL --> D	SHL --> SHL
55	0.00219	0.95297	0.04481	0.00002	0.00001	0.00219	0.94158	0.05600	0.00024	0.00219	0.80446	0.19335	0.00219	0.99781
56	0.00242	0.95274	0.04481	0.00002	0.00001	0.00242	0.94135	0.05600	0.00024	0.00242	0.80423	0.19335	0.00242	0.99758
57	0.00270	0.96143	0.03585	0.00002	0.00001	0.00270	0.95231	0.04480	0.00019	0.00270	0.84261	0.15468	0.00270	0.99730
58	0.00301	0.96112	0.03585	0.00002	0.00001	0.00301	0.95200	0.04480	0.00019	0.00301	0.84231	0.15468	0.00301	0.99699
59	0.00321	0.96092	0.03585	0.00002	0.00001	0.00321	0.95181	0.04480	0.00019	0.00321	0.84211	0.15468	0.00321	0.99679
60	0.00351	0.96061	0.03585	0.00002	0.00001	0.00351	0.95150	0.04480	0.00019	0.00351	0.84180	0.15468	0.00351	0.99649
61	0.00387	0.98716	0.00896	0.00000	0.00000	0.00387	0.98488	0.01120	0.00005	0.00387	0.95746	0.03867	0.00387	0.99613
62	0.00429	0.98674	0.00896	0.00000	0.00000	0.00429	0.98447	0.01120	0.00005	0.00429	0.95704	0.03867	0.00429	0.99571
63	0.00468	0.98635	0.00896	0.00000	0.00000	0.00468	0.98407	0.01120	0.00005	0.00468	0.95665	0.03867	0.00468	0.99532
64	0.00512	0.98592	0.00896	0.00000	0.00000	0.00512	0.98364	0.01120	0.00005	0.00512	0.95621	0.03867	0.00512	0.99488
65	0.00545	0.98559	0.00896	0.00000	0.00000	0.00545	0.98331	0.01120	0.00005	0.00545	0.95588	0.03867	0.00545	0.99455
66	0.00601	0.98502	0.00896	0.00000	0.00000	0.00601	0.98274	0.01120	0.00005	0.00601	0.95532	0.03867	0.00601	0.99399
67	0.00655	0.98448	0.00896	0.00000	0.00000	0.00655	0.98220	0.01120	0.00005	0.00655	0.95478	0.03867	0.00655	0.99345
68	0.00731	0.98373	0.00896	0.00000	0.00000	0.00731	0.98145	0.01120	0.00005	0.00731	0.95402	0.03867	0.00731	0.99269
69	0.00811	0.98292	0.00896	0.00000	0.00000	0.00811	0.98064	0.01120	0.00005	0.00811	0.95322	0.03867	0.00811	0.99189
70	0.00912	0.97563	0.01524	0.00001	0.00000	0.00912	0.97176	0.01904	0.00008	0.00912	0.92514	0.06574	0.00912	0.99088
71	0.00976	0.97500	0.01524	0.00001	0.00000	0.00976	0.97112	0.01904	0.00008	0.00976	0.92450	0.06574	0.00976	0.99024
72	0.01084	0.97391	0.01524	0.00001	0.00000	0.01084	0.97004	0.01904	0.00008	0.01084	0.92342	0.06574	0.01084	0.98916
73	0.01218	0.97258	0.01524	0.00001	0.00000	0.01218	0.96870	0.01904	0.00008	0.01218	0.92208	0.06574	0.01218	0.98782
74	0.01400	0.97076	0.01524	0.00001	0.00000	0.01400	0.96688	0.01904	0.00008	0.01400	0.92026	0.06574	0.01400	0.98600
75	0.01570	0.96905	0.01524	0.00001	0.00000	0.01570	0.96518	0.01904	0.00008	0.01570	0.91856	0.06574	0.01570	0.98430
76	0.01820	0.96655	0.01524	0.00001	0.00000	0.01820	0.96268	0.01904	0.00008	0.01820	0.91606	0.06574	0.01820	0.98180
77	0.02038	0.96437	0.01524	0.00001	0.00000	0.02038	0.96050	0.01904	0.00008	0.02038	0.91388	0.06574	0.02038	0.97962
78	0.02297	0.96178	0.01524	0.00001	0.00000	0.02297	0.95791	0.01904	0.00008	0.02297	0.91129	0.06574	0.02297	0.97703
79	0.02621	0.95855	0.01524	0.00001	0.00000	0.02621	0.95467	0.01904	0.00008	0.02621	0.90805	0.06574	0.02621	0.97379
80	0.02961	0.95515	0.01524	0.00001	0.00000	0.02961	0.95127	0.01904	0.00008	0.02961	0.90465	0.06574	0.02961	0.97039

81	0.03409	0.95066	0.01524	0.00001	0.00000	0.03409	0.94679	0.01904	0.00008	0.03409	0.90017	0.06574	0.03409	0.96591
82	0.03963	0.94513	0.01524	0.00001	0.00000	0.03963	0.94125	0.01904	0.00008	0.03963	0.89463	0.06574	0.03963	0.96037
83	0.04634	0.93841	0.01524	0.00001	0.00000	0.04634	0.93454	0.01904	0.00008	0.04634	0.88792	0.06574	0.04634	0.95366
84	0.05367	0.93108	0.01524	0.00001	0.00000	0.05367	0.92721	0.01904	0.00008	0.05367	0.88059	0.06574	0.05367	0.94633
85	0.06176	0.91842	0.01981	0.00001	0.00000	0.06176	0.91339	0.02475	0.00010	0.06176	0.86593	0.07231	0.06176	0.93824
86	0.07240	0.90778	0.01981	0.00001	0.00000	0.07240	0.90275	0.02475	0.00010	0.07240	0.85529	0.07231	0.07240	0.92760
87	0.08348	0.89670	0.01981	0.00001	0.00000	0.08348	0.89166	0.02475	0.00010	0.08348	0.84420	0.07231	0.08348	0.91652
88	0.09522	0.88496	0.01981	0.00001	0.00000	0.09522	0.87992	0.02475	0.00010	0.09522	0.83246	0.07231	0.09522	0.90478
89	0.10983	0.87035	0.01981	0.00001	0.00000	0.10983	0.86531	0.02475	0.00010	0.10983	0.81785	0.07231	0.10983	0.89017
90	0.12444	0.85574	0.01981	0.00001	0.00000	0.12444	0.85070	0.02475	0.00010	0.12444	0.80325	0.07231	0.12444	0.87556
91	0.14111	0.83908	0.01981	0.00001	0.00000	0.14111	0.83404	0.02475	0.00010	0.14111	0.78658	0.07231	0.14111	0.85889
92	0.15944	0.82074	0.01981	0.00001	0.00000	0.15944	0.81571	0.02475	0.00010	0.15944	0.76825	0.07231	0.15944	0.84056
93	0.17835	0.80183	0.01981	0.00001	0.00000	0.17835	0.79679	0.02475	0.00010	0.17835	0.74933	0.07231	0.17835	0.82165
94	0.19695	0.78323	0.01981	0.00001	0.00000	0.19695	0.77820	0.02475	0.00010	0.19695	0.73074	0.07231	0.19695	0.80305
95	0.22041	0.75581	0.02377	0.00001	0.00001	0.22041	0.74976	0.02970	0.00012	0.22041	0.70727	0.07231	0.22041	0.77959
96	0.24374	0.73247	0.02377	0.00001	0.00001	0.24374	0.72643	0.02970	0.00012	0.24374	0.68394	0.07231	0.24374	0.75626
97	0.26032	0.71590	0.02377	0.00001	0.00001	0.26032	0.70986	0.02970	0.00012	0.26032	0.66737	0.07231	0.26032	0.73968
98	0.27684	0.69938	0.02377	0.00001	0.00001	0.27684	0.69333	0.02970	0.00012	0.27684	0.65085	0.07231	0.27684	0.72316
99	0.29884	0.67737	0.02377	0.00001	0.00001	0.29884	0.67133	0.02970	0.00012	0.29884	0.62884	0.07231	0.29884	0.70116
100	0.32976	0.64646	0.02377	0.00001	0.00001	0.32976	0.64042	0.02970	0.00012	0.32976	0.59793	0.07231	0.32976	0.67024
101	0.37285	0.60337	0.02377	0.00001	0.00001	0.37285	0.59732	0.02970	0.00012	0.37285	0.55483	0.07231	0.37285	0.62715
102	0.40390	0.57231	0.02377	0.00001	0.00001	0.40390	0.56627	0.02970	0.00012	0.40390	0.52378	0.07231	0.40390	0.59610
103	0.43572	0.54050	0.02377	0.00001	0.00001	0.43572	0.53446	0.02970	0.00012	0.43572	0.49197	0.07231	0.43572	0.56428
104	0.46804	0.50818	0.02377	0.00001	0.00001	0.46804	0.50213	0.02970	0.00012	0.46804	0.45964	0.07231	0.46804	0.53196
105	0.50016	0.47606	0.02377	0.00001	0.00001	0.50016	0.47001	0.02970	0.00012	0.50016	0.42753	0.07231	0.50016	0.49984
106	0.53267	0.44354	0.02377	0.00001	0.00001	0.53267	0.43750	0.02970	0.00012	0.53267	0.39501	0.07231	0.53267	0.46733
107	0.56489	0.41132	0.02377	0.00001	0.00001	0.56489	0.40528	0.02970	0.00012	0.56489	0.36279	0.07231	0.56489	0.43511
108	0.59656	0.37966	0.02377	0.00001	0.00001	0.59656	0.37361	0.02970	0.00012	0.59656	0.33113	0.07231	0.59656	0.40344
109	0.62744	0.34878	0.02377	0.00001	0.00001	0.62744	0.34273	0.02970	0.00012	0.62744	0.30024	0.07231	0.62744	0.37256

Key. D = death, MIHL = mild hearing loss, MOHL = moderate hearing loss, NH = normal hearing, SHL = severe hearing loss

Review of published economic evaluations (more details)

In the health economics and outcomes research literature, few examples of cost-utility analyses on HAs for age-related HL exists. One of the first examples is the work by Boas and colleagues[2] who estimated an incremental cost-utility ratio (ICUR) of € 18,046/quality-adjusted life year (QALY) for HA plus post-purchase service compared to no treatment and € 21,154/QALY for HA use alone compared to no treatment using a dynamic decision model applied to the Dutch healthcare system. Another work based on the Dutch context is the one by Joore and coauthors[3], who estimated 0.03 incremental QALYs gained and an ICUR of € 15,807/QALY for HA use compared to no treatment using a Markov model; as pointed out by Chao and Chen[4], their work was mainly limited by the inability of the model to distinguish across different disease severity states and it was lacking a proper sensitivity analysis. Chao and Chen[4] advanced the literature on the field by producing a multistate Markov model that considered distinct HL states based on disease severity, modelled explicitly HAs non-use due to dissatisfaction and performed a thorough sensitivity analysis; they estimated ICURs varying from € 7,715/QALY to € 10,826/QALY in the context of the Taiwanese healthcare system, adopting a societal perspective. Finally, the recent work by Mandavia and colleagues[5] focused on novel hearing therapeutics applied to idiopathic sudden sensorineural HL – still in the context of presbycusis – estimating an incremental net monetary benefit (INMB) of £39,032 from the perspective of the British National Health Service (NHS).

Patient journey completion, hearing aids prescription, hearing aids purchase and compliance probabilities (more details)

Patient journey completion was defined as the successful completion of the following three events: i) the hearing impaired patient discussed HL with ear doctor and/or family doctor, ii) the hearing impaired patient received positive advice (ear doctor or family doctor recommended further action), and iii) the hearing impaired patient discussed HL with a HAs dispenser or audiologist. Estimates implemented in the model were computed as conditional probabilities; for example: Eurotrak 2018[6] reports that, out of 100 hearing impaired

patients belonging to the top 50% HL subsample, 54 discussed HL with ear doctor and/or family doctor, received positive advice (ear doctor or family doctor recommended further action), and discussed HL with a HAs dispenser or audiologist; among these, 50 received positive HA advice from dispenser / audiologist and 47 bought a HA. As a consequence, a probability of 54% for patient journey completion, 93% for receiving HA prescription, and 84% for HA purchase were imputed in the model when patients transitioned to severe HL state from a previously unaided state. As for the probability of being compliant, no stratified data were available; as a consequence, the estimate (94%) was applied irrespectively of HL severity; this approach was deemed realistic also based on empirical data showing that adherence in Italy is the highest among all countries in which Eurotrak surveys are administered[6].

The literature emphasizes the importance of audiologists and hearing care professionals in increasing the likelihood of compliance in patients through the customization of the device also based on patients' lifestyle and hearing needs[7]; this aspect was captured in the model by distinguishing HA use plus post-purchase service and HA use alone as two separate intervention arms. In order to distinguish the impact on compliance between HA plus post-purchase service and HA alone, a 36% penalization on the probability of compliance was applied to the HA alone arm based on the approach used by Boas and colleagues[2].

Quality of life improvement after HA use (more details)

The first set of utility weights relies on the estimates of utility weights assigned to grades of HL by Shield[8] (in turn based on Barton et al.[9], Davis et al.[10], and Swan et al.[11]), assuming increments in QoL for HAs use based on Chao and Chen[4] corrected for clinical plausibility (e.g., the utility increment from unaided moderate HL to aided moderate HL was deemed excessively large and it was modified to maintain monotonicity, i.e., so that the utility weight attached to aided mild HL being at least equal or larger than the one attached to aided moderate HL). The second one relies on strategy proposed by Mandavia and colleagues[5], using estimates from Linssen et al.[12] and Arndt et al.[13]. The main difference between the two sets is that, being based on different instruments for QoL estimation, the former attaches lower utility weights for HL states with respect to the latter. In both cases it was assumed a utility weight of 1 for normal hearing, as in Chao and Chen[4].

Costs (more details)

The model allows to distinguish between the costs of specialists visits delivered through the Italian NHS, estimated using Italian published sources (*Nomenclatore dell'assistenza specialistica ambulatoriale*[14]) and the ones delivered privately, estimated based on expert opinion. In the base case, a weighted average of the two delivery systems using equal weights was assumed due to lack of reliable estimates on the real-world proportions. It is worth noticing, however, that specialist visits delivered through the Italian NHS usually require longer waiting times compared to private providers and this aspect, which can alter the decision-making process of patients; for this reason, the parameter was stressed in the sensitivity analysis. The cost of the device is applied when patients buy it and, in case they are compliant, it is assumed to be replaced each five years. Real world data[15] show that the Italian HAs market can be subdivided into three categories: 20% is entirely covered by the Italian NHS (*mercato sociale*), 26% is based on copayment (*mercato riconducibile*), and the remaining proportion is purely private (*mercato libero*). As a consequence, in the base case the cost of the device was estimated using the weighted average between the cost reimbursed by the NHS (based on the tariff published on *Nomenclatore tariffario D.M. 332/1999*[16]) and the price applied by the private suppliers. As for the cost of the device, it has to be recognized that several categories of HAs coexist in the market, differing by degree of sophistication of the features offered; in order to achieve a realistic representation of the Italian context, the price associated to the "standard" class of HAs, i.e., the one accounting for the largest market share according to expert opinion, was used.

As for unintentional falls in older people, data on age-specific annual incidence of unintentional falls in older people leading to accident and emergency attendances was retrieved from Scuffham and colleagues[17] and were used as baseline probabilities to which the odds ratios for injurious falls leading to hospitalization in unaided and aided hearing impaired elderly individuals were applied (Girard et al.[18] for the former, Mahmoudi et al.[19] for the latter).

As for productivity losses, it is recognized in the literature that HL can negatively impact on employment and career opportunity, and the related costs were estimated to account for 18.6% of total costs[20]. A peer-validated approach to estimate productivity losses consists in applying an 18% employment gap between

moderately-to-severely hearing impaired individuals and normal-hearing individuals aged at most 64 years in countries which have full employment (operationalized as unemployment rate below 6%). However, based on the most updated employment rates available[21], Italy does not satisfy the full employment criterion. As a consequence, in order to account for productivity losses, the estimate based on real world data of $48 per 7 months as excess occupational health expenditure by Nachtegaal and colleagues[22] was applied to unaided moderate and severe HL states up to 64 years of age; as a proxy for the mitigating effect of HA use, a reduction equal to the percentage difference between unemployment rates in unaided and aided hearing impaired individuals estimated by Kochkin[23] was used.

Sensitivity analysis

Table 7 Deterministic Sensitivity Analysis (DSA) parameters

Parameter	Variation interval
**Pathway probabilities**	
Probability of patient journey completion, mild HL	±5%
Probability of patient journey completion, moderate HL	±5%
Probability of patient journey completion, severe HL	±5%
Probability of HA prescription, mild HL	±5%
Probability of HA prescription, moderate HL	±5%
Probability of HA prescription, severe HL	±5%
Probability of HA purchase, mild HL	±5%
Probability of HA purchase, moderate HL	±5%
Probability of HA purchase, severe HL	±5%
Probability of compliance with post-purchase service, mild HL	±5%
Probability of compliance with post-purchase service, moderate HL	±5%
Probability of compliance with post-purchase service, severe HL	±5%
Expected reduction in compliance in absence of post-purchase service	±10%
**Health utilities**	
Mild HL, unaided	±10%
Moderate HL, unaided	±10%
Severe HL, unaided	±10%
Mild HL, aided	±10%
Moderate HL, aided	±10%
Severe HL, aided	±10%
**Costs**	

Price of the device in the private market	±10%
Public reimbursement tariff for the device	±10%
Transportation costs	±10%
Daily productivity loss (daily gross national product per capita)	±10%
ENT first visit, NHS	±10%
ENT first visit, private healthcare provider	±10%
ENT follow-up visit, NHS	±10%
ENT follow-up visit, private healthcare provider	±10%
**Other parameters**	
Proportion of specialist visits offered through the NHS	±10%
Proportion of fully reimbursed devices	±10%
Proportion of partially reimbursed devices (copayment)	±10%

Key. ENT = ears, nose and throat doctor; HA = hearing aid; HL = hearing loss; NHS = national healthcare system

Scenario analysis results

Base-case public-private mix, utilities Shield 2018[8]
Current dropout and compliance mix

Gender: male

**Outcome**	**HA + pps**	**HA**
Mean costs (€)	2,260	2,187
Mean LYs	18.4096	18.4096
Mean QALYs	17.4070	17.3445
NMB	280,865	279,921
**Compared with HA w/out post-purchase service**		
Incremental costs (€)	73	
Incremental QALYs	0.0625	
ICUR (€/QALYs)	1,166	
INMB	944	

**Outcome**	**HA + pps**	**No treat**
Mean costs (€)	2,260	1,909
Mean LYs	18.4096	18.4096
Mean QALYs	17.4070	17.2284
NMB	280,865	278,311
**Compared with no treatment**		
Incremental costs (€)	351	
Incremental QALYs	0.1786	
ICUR (€/QALYs)	1,966	
INMB	2,554	

Gender: female

**Outcome**	**HA + pps**	**HA**
Mean costs (€)	2,674	2,599
Mean LYs	20.0094	20.0094
Mean QALYs	18.9073	18.8416
NMB	304,854	303,859
**Compared with HA w/out post-purchase service**		
Incremental costs (€)	75	
Incremental QALYs	0.0657	
ICUR (€/QALYs)	1,135	
INMB	995	

**Outcome**	**HA + pps**	**No treat**
Mean costs (€)	2,674	2,288
Mean LYs	20.0094	20.0094
Mean QALYs	18.9073	18.7132
NMB	304,854	302,082
**Compared with no treatment**		
Incremental costs (€)	385	
Incremental QALYs	0.1941	
ICUR (€/QALYs)	1,986	
INMB	2,772	

Outcome	HA	No treat
Mean costs (€)	2,187	1,909
Mean LYs	18.4096	18.4096
Mean QALYs	17.3445	17.2284
NMB	279,921	278,311
**Compared with no treatment**		
Incremental costs (€)	278	
Incremental QALYs	0.1161	
ICUR (€/QALYs)	2,397	
INMB	1,610	

Outcome	HA	No treat
Mean costs (€)	2,599	2,288
Mean LYs	20.0094	20.0094
Mean QALYs	18.8416	18.7132
NMB	303,859	302,082
**Compared with no treatment**		
Incremental costs (€)	311	
Incremental QALYs	0.1284	
ICUR (€/QALYs)	2,422	
INMB	1,777	

No dropout, partial compliance

Gender: male

Outcome	HA + pps	HA
Mean costs (€)	3,568	3,305
Mean LYs	18.4096	18.4096
Mean QALYs	18.0006	17.7898
NMB	289,212	286,047
**Compared with HA w/out post-purchase service**		
Incremental costs (€)	263	
Incremental QALYs	0.2108	
ICUR (€/QALYs)	1,248	
INMB	3,165	

Gender: female

Outcome	HA + pps	HA
Mean costs (€)	4,052	3,753
Mean LYs	20.0094	20.0094
Mean QALYs	19.4988	19.2814
NMB	313,097	309,860
**Compared with HA w/out post-purchase service**		
Incremental costs (€)	299	
Incremental QALYs	0.2174	
ICUR (€/QALYs)	1,375	
INMB	3,237	

Outcome	HA + pps	No treat
Mean costs (€)	3,568	1,909
Mean LYs	18.4096	18.4096
Mean QALYs	18.0006	17.2284
NMB	289,212	278,311
**Compared with no treatment**		
Incremental costs (€)	1,659	
Incremental QALYs	0.7722	
ICUR (€/QALYs)	2,148	
INMB	10,901	

Outcome	HA + pps	No treat
Mean costs (€)	4,052	2,288
Mean LYs	20.0094	20.0094
Mean QALYs	19.4988	18.7132
NMB	313,097	302,082
**Compared with no treatment**		
Incremental costs (€)	1,763	
Incremental QALYs	0.7856	
ICUR (€/QALYs)	2,245	
INMB	11,015	

Outcome	HA	No treat
Mean costs (€)	3,305	1,909
Mean LYs	18.4096	18.4096
Mean QALYs	17.7898	17.2284
NMB	286,047	278,311
**Compared with no treatment**		
Incremental costs (€)	1,396	
Incremental QALYs	0.5614	
ICUR (€/QALYs)	2,487	
INMB	7,736	

Outcome	HA	No treat
Mean costs (€)	3,753	2,288
Mean LYs	20.0094	20.0094
Mean QALYs	19.2814	18.7132
NMB	309,860	302,082
**Compared with no treatment**		
Incremental costs (€)	1,464	
Incremental QALYs	0.5682	
ICUR (€/QALYs)	2,577	
INMB	7,778	

No dropout, full compliance

Gender: male

Outcome	HA + pps	HA
Mean costs (€)	3,609	3,338
Mean LYs	18.4096	18.4096
Mean QALYs	18.0301	17.8176
NMB	289,651	286,465
**Compared with HA w/out post-purchase service**		
Incremental costs (€)	271	
Incremental QALYs	0.2125	
ICUR (€/QALYs)	1,276	
INMB	3,186	

Outcome	HA + pps	No treat
Mean costs (€)	3,609	1,909
Mean LYs	18.4096	18.4096
Mean QALYs	18.0301	17.2284
NMB	289,651	278,311
**Compared with no treatment**		
Incremental costs (€)	1,700	
Incremental QALYs	0.8017	
ICUR (€/QALYs)	2,120	
INMB	11,340	

Gender: female

Outcome	HA + pps	HA
Mean costs (€)	4,103	3,788
Mean LYs	20.0094	20.0094
Mean QALYs	19.5321	19.3091
NMB	313,586	310,274
**Compared with HA w/out post-purchase service**		
Incremental costs (€)	315	
Incremental QALYs	0.2230	
ICUR (€/QALYs)	1,413	
INMB	3,312	

Outcome	HA + pps	No treat
Mean costs (€)	4,103	2,288
Mean LYs	20.0094	20.0094
Mean QALYs	19.5321	18.7132
NMB	313,586	302,082
**Compared with no treatment**		
Incremental costs (€)	1,815	
Incremental QALYs	0.8189	
ICUR (€/QALYs)	2,216	
INMB	11,504	

Outcome	HA	No treat
Mean costs (€)	3,338	1,909
Mean LYs	18.4096	18.4096
Mean QALYs	17.8176	17.2284
NMB	286,465	278,311
**Compared with no treatment**		
Incremental costs (€)	1,429	
Incremental QALYs	0.5891	
ICUR (€/QALYs)	2,425	
INMB	8,154	

Outcome	HA	No treat
Mean costs (€)	3,788	2,288
Mean LYs	20.0094	20.0094
Mean QALYs	19.3091	18.7132
NMB	310,274	302,082
**Compared with no treatment**		
Incremental costs (€)	1,499	
Incremental QALYs	0.5959	
ICUR (€/QALYs)	2,517	
INMB	8,192	

Base-case public-private mix, utilities Mandavia et al., 2020[5]

Current dropout and compliance mix

Gender: male

Outcome	HA + pps	HA
Mean costs (€)	2,260	2,187
Mean LYs	18.4096	18.4096
Mean QALYs	17.5216	17.4926
NMB	282,729	282,330
**Compared with HA w/out post-purchase service**		
Incremental costs (€)	73	
Incremental QALYs	0.0290	
ICUR (€/QALYs)	2,512	
INMB	399	

Gender: female

Outcome	HA + pps	HA
Mean costs (€)	2,674	2,599
Mean LYs	20.0094	20.0094
Mean QALYs	19.0041	18.9712
NMB	306,429	305,967
**Compared with HA w/out post-purchase service**		
Incremental costs (€)	75	
Incremental QALYs	0.0329	
ICUR (€/QALYs)	2,264	
INMB	461	

Outcome	HA + pps	No treat
Mean costs (€)	2,260	1,909
Mean LYs	18.4096	18.4096
Mean QALYs	17.5216	17.4394
NMB	282,729	281,742
**Compared with no treatment**		
Incremental costs (€)	351	
Incremental QALYs	0.0822	
ICUR (€/QALYs)	4,270	
INMB	986	

Outcome	HA + pps	No treat
Mean costs (€)	2,674	2,288
Mean LYs	20.0094	20.0094
Mean QALYs	19.0041	18.9084
NMB	306,429	305,257
**Compared with no treatment**		
Incremental costs (€)	385	
Incremental QALYs	0.0957	
ICUR (€/QALYs)	4,028	
INMB	1,171	

Outcome	HA	No treat
Mean costs (€)	2,187	1,909
Mean LYs	18.4096	18.4096
Mean QALYs	17.4926	17.4394
NMB	282,330	281,742
**Compared with no treatment**		
Incremental costs (€)	278	
Incremental QALYs	0.0532	
ICUR (€/QALYs)	5,229	
INMB	587	

Outcome	HA	No treat
Mean costs (€)	2,599	2,288
Mean LYs	20.0094	20.0094
Mean QALYs	18.9712	18.9084
NMB	305,967	305,257
**Compared with no treatment**		
Incremental costs (€)	311	
Incremental QALYs	0.0628	
ICUR (€/QALYs)	4,954	
INMB	710	

No dropout, partial compliance

Gender: male

Outcome	HA + pps	HA
Mean costs (€)	3,568	3,305
Mean LYs	18.4096	18.4096
Mean QALYs	17.8372	17.7157
NMB	286,554	284,840
**Compared with HA w/out post-purchase service**		
Incremental costs (€)	263	
Incremental QALYs	0.1215	
ICUR (€/QALYs)	2,164	
INMB	1,714	

Outcome	HA + pps	No treat
Mean costs (€)	3,568	1,909
Mean LYs	18.4096	18.4096
Mean QALYs	17.8372	17.4394
NMB	286,554	281,742
**Compared with no treatment**		
Incremental costs (€)	1,659	
Incremental QALYs	0.3978	
ICUR (€/QALYs)	4,170	
INMB	4,812	

Gender: female

Outcome	HA + pps	HA
Mean costs (€)	4,052	3,753
Mean LYs	20.0094	20.0094
Mean QALYs	19.3530	19.2156
NMB	310,725	308,790
**Compared with HA w/out post-purchase service**		
Incremental costs (€)	299	
Incremental QALYs	0.1374	
ICUR (€/QALYs)	2,177	
INMB	1,936	

Outcome	HA + pps	No treat
Mean costs (€)	4,052	2,288
Mean LYs	20.0094	20.0094
Mean QALYs	19.3530	18.9084
NMB	310,725	305,257
**Compared with no treatment**		
Incremental costs (€)	1,763	
Incremental QALYs	0.4446	
ICUR (€/QALYs)	3,967	
INMB	5,468	

Outcome	HA	No treat
Mean costs (€)	3,305	1,909
Mean LYs	18.4096	18.4096
Mean QALYs	17.7157	17.4394
NMB	284,840	281,742
**Compared with no treatment**		
Incremental costs (€)	1,396	
Incremental QALYs	0.2763	
ICUR (€/QALYs)	5,053	
INMB	3,098	

Outcome	HA	No treat
Mean costs (€)	3,753	2,288
Mean LYs	20.0094	20.0094
Mean QALYs	19.2156	18.9084
NMB	308,790	305,257
**Compared with no treatment**		
Incremental costs (€)	1,464	
Incremental QALYs	0.3072	
ICUR (€/QALYs)	4,767	
INMB	3,532	

No dropout, full compliance

Gender: male

Outcome	HA + pps	HA
Mean costs (€)	3,609	3,338
Mean LYs	18.4096	18.4096
Mean QALYs	17.8562	17.7307
NMB	286,822	285,052
**Compared with HA w/out post-purchase service**		
Incremental costs (€)	271	
Incremental QALYs	0.1255	
ICUR (€/QALYs)	2,160	
INMB	1,771	

Gender: female

Outcome	HA + pps	HA
Mean costs (€)	4,103	3,788
Mean LYs	20.0094	20.0094
Mean QALYs	19.3756	19.2322
NMB	311,041	309,024
**Compared with HA w/out post-purchase service**		
Incremental costs (€)	315	
Incremental QALYs	0.1434	
ICUR (€/QALYs)	2,198	
INMB	2,017	

Outcome	HA + pps	No treat
Mean costs (€)	3,609	1,909
Mean LYs	18.4096	18.4096
Mean QALYs	17.8562	17.4394
NMB	286,822	281,742
**Compared with no treatment**		
Incremental costs (€)	1,700	
Incremental QALYs	0.4168	
ICUR (€/QALYs)	4,078	
INMB	5,080	

Outcome	HA + pps	No treat
Mean costs (€)	4,103	2,288
Mean LYs	20.0094	20.0094
Mean QALYs	19.3756	18.9084
NMB	311,041	305,257
**Compared with no treatment**		
Incremental costs (€)	1,815	
Incremental QALYs	0.4672	
ICUR (€/QALYs)	3,884	
INMB	5,784	

Outcome	HA	No treat
Mean costs (€)	3,338	1,909
Mean LYs	18.4096	18.4096
Mean QALYs	17.7307	17.4394
NMB	285,052	281,742
**Compared with no treatment**		
Incremental costs (€)	1,429	
Incremental QALYs	0.2913	
ICUR (€/QALYs)	4,905	
INMB	3,309	

Outcome	HA	No treat
Mean costs (€)	3,788	2,288
Mean LYs	20.0094	20.0094
Mean QALYs	19.2322	18.9084
NMB	309,024	305,257
**Compared with no treatment**		
Incremental costs (€)	1,499	
Incremental QALYs	0.3238	
ICUR (€/QALYs)	4,631	
INMB	3,767	

Private prices only, utilities Shield 2018

Current dropout and compliance mix

Gender: male

Outcome	HA + pps	HA
Mean costs (€)	2,403	2,288
Mean LYs	18.4096	18.4096
Mean QALYs	17.4070	17.3445
NMB	280,723	279,820
**Compared with HA w/out post-purchase service**		
Incremental costs (€)	114	
Incremental QALYs	0.0625	
ICUR (€/QALYs)	1,828	
INMB	902	

Outcome	HA + pps	No treat
Mean costs (€)	2,403	1,909
Mean LYs	18.4096	18.4096
Mean QALYs	17.4070	17.2284
NMB	280,723	278,311
**Compared with no treatment**		
Incremental costs (€)	493	
Incremental QALYs	0.1786	
ICUR (€/QALYs)	2,763	
INMB	2,412	

Gender: female

Outcome	HA + pps	HA
Mean costs (€)	2,838	2,717
Mean LYs	20.0094	20.0094
Mean QALYs	18.9073	18.8416
NMB	304,690	303,742
**Compared with HA w/out post-purchase service**		
Incremental costs (€)	121	
Incremental QALYs	0.0657	
ICUR (€/QALYs)	1,839	
INMB	948	

Outcome	HA + pps	No treat
Mean costs (€)	2,838	2,288
Mean LYs	20.0094	20.0094
Mean QALYs	18.9073	18.7132
NMB	304,690	302,082
**Compared with no treatment**		
Incremental costs (€)	549	
Incremental QALYs	0.1941	
ICUR (€/QALYs)	2,829	
INMB	2,608	

Outcome	HA	No treat
Mean costs (€)	2,288	1,909
Mean LYs	18.4096	18.4096
Mean QALYs	17.3445	17.2284
NMB	279,820	278,311
**Compared with no treatment**		
Incremental costs (€)	379	
Incremental QALYs	0.1161	
ICUR (€/QALYs)	3,266	
INMB	1,509	

Outcome	HA	No treat
Mean costs (€)	2,717	2,288
Mean LYs	20.0094	20.0094
Mean QALYs	18.8416	18.7132
NMB	303,742	302,082
**Compared with no treatment**		
Incremental costs (€)	428	
Incremental QALYs	0.1284	
ICUR (€/QALYs)	3,336	
INMB	1,660	

No dropout, partial compliance

Gender: male

Outcome	HA + pps	HA
Mean costs (€)	4,240	3,813
Mean LYs	18.4096	18.4096
Mean QALYs	18.0006	17.7898
NMB	288,540	285,538
**Compared with HA w/out post-purchase service**		
Incremental costs (€)	426	
Incremental QALYs	0.2108	
ICUR (€/QALYs)	2,023	
INMB	3,002	

Gender: female

Outcome	HA + pps	HA
Mean costs (€)	4,797	4,309
Mean LYs	20.0094	20.0094
Mean QALYs	19.4988	19.2814
NMB	312,352	309,303
**Compared with HA w/out post-purchase service**		
Incremental costs (€)	488	
Incremental QALYs	0.2174	
ICUR (€/QALYs)	2,244	
INMB	3048.192194	

Outcome	HA + pps	No treat
Mean costs (€)	4,240	1,909
Mean LYs	18.4096	18.4096
Mean QALYs	18.0006	17.2284
NMB	288,540	278,311
**Compared with no treatment**		
Incremental costs (€)	2,331	
Incremental QALYs	0.7722	
ICUR (€/QALYs)	3,018	
INMB	10,229	

Outcome	HA + pps	No treat
Mean costs (€)	4,797	2,288
Mean LYs	20.0094	20.0094
Mean QALYs	19.4988	18.7132
NMB	312351.6	302,082
**Compared with no treatment**		
Incremental costs (€)	2,509	
Incremental QALYs	0.7856	
ICUR (€/QALYs)	3,193	
INMB	10,270	

Outcome	HA	No treat
Mean costs (€)	3,813	1,909
Mean LYs	18.4096	18.4096
Mean QALYs	17.7898	17.2284
NMB	285,538	278,311
**Compared with no treatment**		
Incremental costs (€)	1,904	
Incremental QALYs	0.5614	
ICUR (€/QALYs)	3,392	
INMB	7,227	

Outcome	HA	No treat
Mean costs (€)	4,309	2,288
Mean LYs	20.0094	20.0094
Mean QALYs	19.2814	18.7132
NMB	309,303	302,082
**Compared with no treatment**		
Incremental costs (€)	2,021	
Incremental QALYs	0.5682	
ICUR (€/QALYs)	3,556	
INMB	7,222	

No dropout, full compliance

Gender: male

**Outcome**	**HA + pps**	**HA**
Mean costs (€)	4,306	3,866
Mean LYs	18.4096	18.4096
Mean QALYs	18.0301	17.8176
NMB	288,954	285,936
**Compared with HA w/out post-purchase service**		
Incremental costs (€)	439	
Incremental QALYs	0.2125	
ICUR (€/QALYs)	2,067	
INMB	3,018	

**Outcome**	**HA + pps**	**No treat**
Mean costs (€)	4,306	1,909
Mean LYs	18.4096	18.4096
Mean QALYs	18.0301	17.2284
NMB	288,954	278,311
**Compared with no treatment**		
Incremental costs (€)	2,397	
Incremental QALYs	0.8017	
ICUR (€/QALYs)	2,990	
INMB	10,643	

Gender: female

**Outcome**	**HA + pps**	**HA**
Mean costs (€)	4,880	4,367
Mean LYs	20.0094	20.0094
Mean QALYs	19.5321	19.3091
NMB	312,810	309,695
**Compared with HA w/out post-purchase service**		
Incremental costs (€)	513	
Incremental QALYs	0.2230	
ICUR (€/QALYs)	2,299	
INMB	3,114	

**Outcome**	**HA + pps**	**No treat**
Mean costs (€)	4,880	2,288
Mean LYs	20.0094	20.0094
Mean QALYs	19.5321	18.7132
NMB	312,810	302,082
**Compared with no treatment**		
Incremental costs (€)	2,591	
Incremental QALYs	0.8189	
ICUR (€/QALYs)	3,164	
INMB	10,728	

Outcome	HA	No treat
Mean costs (€)	3,866	1,909
Mean LYs	18.4096	18.4096
Mean QALYs	17.8176	17.2284
NMB	285,936	278,311
**Compared with no treatment**		
Incremental costs (€)	1,957	
Incremental QALYs	0.5891	
ICUR (€/QALYs)	3,322	
INMB	7,625	

Outcome	HA	No treat
Mean costs (€)	4,367	2,288
Mean LYs	20.0094	20.0094
Mean QALYs	19.3091	18.7132
NMB	309,695	302,082
**Compared with no treatment**		
Incremental costs (€)	2,078	
Incremental QALYs	0.5959	
ICUR (€/QALYs)	3,488	
INMB	7,613	

Private prices only, utilities Mandavia et al., 2020

Current dropout and compliance mix

Gender: male

Outcome	HA + pps	HA
Mean costs (€)	2,403	2,288
Mean LYs	18.4096	18.4096
Mean QALYs	17.5216	17.4926
NMB	282,587	282,229
**Compared with HA w/out post-purchase service**		
Incremental costs (€)	114	
Incremental QALYs	0.0290	
ICUR (€/QALYs)	3,937	

Gender: female

Outcome	HA + pps	HA
Mean costs (€)	2,838	2,717
Mean LYs	20.0094	20.0094
Mean QALYs	19.0041	18.9712
NMB	306,265	305,850
**Compared with HA w/out post-purchase service**		
Incremental costs (€)	121	
Incremental QALYs	0.0329	
ICUR (€/QALYs)	3,669	

INMB	358		INMB	415

Outcome	HA + pps	No treat
Mean costs (€)	2,403	1,909
Mean LYs	18.4096	18.4096
Mean QALYs	17.5216	17.4394
NMB	282,587	281,742
**Compared with no treatment**		
Incremental costs (€)	493	
Incremental QALYs	0.0822	
ICUR (€/QALYs)	6,000	
INMB	844	

Outcome	HA + pps	No treat
Mean costs (€)	2,838	2,288
Mean LYs	20.0094	20.0094
Mean QALYs	19.0041	18.9084
NMB	306,265	305,257
**Compared with no treatment**		
Incremental costs (€)	549	
Incremental QALYs	0.0957	
ICUR (€/QALYs)	5,739	
INMB	1,007	

Outcome	HA	No treat
Mean costs (€)	2,288	1,909
Mean LYs	18.4096	18.4096
Mean QALYs	17.4926	17.4394
NMB	282,229	281,742
**Compared with no treatment**		
Incremental costs (€)	379	
Incremental QALYs	0.0532	
ICUR (€/QALYs)	7,126	
INMB	486	

Outcome	HA	No treat
Mean costs (€)	2,717	2,288
Mean LYs	20.0094	20.0094
Mean QALYs	18.9712	18.9084
NMB	305,850	305,257
**Compared with no treatment**		
Incremental costs (€)	428	
Incremental QALYs	0.0628	
ICUR (€/QALYs)	6,826	
INMB	592	

No dropout, partial compliance

Gender: male

Outcome	HA + pps	HA
Mean costs (€)	4,240	3,813
Mean LYs	18.4096	18.4096
Mean QALYs	17.8372	17.7157
NMB	285,882	284,332
**Compared with HA w/out post-purchase service**		
Incremental costs (€)	426	
Incremental QALYs	0.1215	
ICUR (€/QALYs)	3,507	
INMB	1,551	

Gender: female

Outcome	HA + pps	HA
Mean costs (€)	4,797	4,309
Mean LYs	20.0094	20.0094
Mean QALYs	19.3530	19.2156
NMB	309,980	308,233
**Compared with HA w/out post-purchase service**		
Incremental costs (€)	488	
Incremental QALYs	0.1374	
ICUR (€/QALYs)	3,552	
INMB	1,747	

Gender: male

Outcome	HA + pps	No treat
Mean costs (€)	4,240	1,909
Mean LYs	18.4096	18.4096
Mean QALYs	17.8372	17.4394
NMB	285,882	281,742
**Compared with no treatment**		
Incremental costs (€)	2,331	
Incremental QALYs	0.3978	
ICUR (€/QALYs)	5,858	

Gender: female

Outcome	HA + pps	No treat
Mean costs (€)	4,797	2,288
Mean LYs	20.0094	20.0094
Mean QALYs	19.3530	18.9084
NMB	309,980	305,257
**Compared with no treatment**		
Incremental costs (€)	2,509	
Incremental QALYs	0.4446	
ICUR (€/QALYs)	5,642	

INMB	4,140		INMB	4,723

Outcome	HA	No treat	Outcome	HA	No treat
Mean costs (€)	3,813	1,909	Mean costs (€)	4,309	2,288
Mean LYs	18.4096	18.4096	Mean LYs	20.0094	20.0094
Mean QALYs	17.7157	17.4394	Mean QALYs	19.2156	18.9084
NMB	284,332	281,742	NMB	308,233	305,257
**Compared with no treatment**			**Compared with no treatment**		
Incremental costs (€)	1,904		Incremental costs (€)	2,021	
Incremental QALYs	0.2763		Incremental QALYs	0.3072	
ICUR (€/QALYs)	6,893		ICUR (€/QALYs)	6,578	
INMB	2,589		INMB	2,976	

No dropout, full compliance

Gender: male | **Gender: female**

Outcome	HA + pps	HA	Outcome	HA + pps	HA
Mean costs (€)	4,306	3,866	Mean costs (€)	4,880	4,367
Mean LYs	18.4096	18.4096	Mean LYs	20.0094	20.0094
Mean QALYs	17.8562	17.7307	Mean QALYs	19.3756	19.2322
NMB	286,125	284,523	NMB	310,265	308,446
**Compared with HA w/out post-purchase service**			**Compared with HA w/out post-purchase service**		
Incremental costs (€)	439		Incremental costs (€)	513	
Incremental QALYs	0.1255		Incremental QALYs	0.1434	

ICUR (€/QALYs)	3,500		ICUR (€/QALYs)	3,577	
INMB	1,602		INMB	1,819	

Outcome	HA + pps	No treat	Outcome	HA + pps	No treat
Mean costs (€)	4,306	1,909	Mean costs (€)	4,880	2,288
Mean LYs	18.4096	18.4096	Mean LYs	20.0094	20.0094
Mean QALYs	17.8562	17.4394	Mean QALYs	19.3756	18.9084
NMB	286,125	281,742	NMB	310,265	305,257
**Compared with no treatment**			**Compared with no treatment**		
Incremental costs (€)	2,397		Incremental costs (€)	2,591	
Incremental QALYs	0.4168		Incremental QALYs	0.4672	
ICUR (€/QALYs)	5,750		ICUR (€/QALYs)	5,546	
INMB	4,383		INMB	5,007	

Outcome	HA	No treat	Outcome	HA	No treat
Mean costs (€)	3,866	1,909	Mean costs (€)	4,367	2,288
Mean LYs	18.4096	18.4096	Mean LYs	20.0094	20.0094
Mean QALYs	17.7307	17.4394	Mean QALYs	19.2322	18.9084
NMB	284,523	281,742	NMB	308,446	305,257
**Compared with no treatment**			**Compared with no treatment**		
Incremental costs (€)	1,957		Incremental costs (€)	2,078	
Incremental QALYs	0.2913		Incremental QALYs	0.3238	
ICUR (€/QALYs)	6,719		ICUR (€/QALYs)	6,419	
INMB	2,781		INMB	3,188	

Public tariffs only, utilities Shield

Current dropout and compliance mix

Gender: male

Outcome	HA + pps	HA
Mean costs (€)	2,046	2,020
Mean LYs	18.4096	18.4096
Mean QALYs	17.4070	17.3445
NMB	281,079	280,089
**Compared with HA w/out post-purchase service**		
Incremental costs (€)	27	
Incremental QALYs	0.0625	
ICUR (€/QALYs)	426	
INMB	990	

Outcome	HA + pps	No treat
Mean costs (€)	2,046	1,909
Mean LYs	18.4096	18.4096
Mean QALYs	17.4070	17.2284
NMB	281,079	278,311

Gender: female

Outcome	HA + pps	HA
Mean costs (€)	2,429	2,405
Mean LYs	20.0094	20.0094
Mean QALYs	18.9073	18.8416
NMB	305,099	304,054
**Compared with HA w/out post-purchase service**		
Incremental costs (€)	24	
Incremental QALYs	0.0657	
ICUR (€/QALYs)	364	
INMB	1,045	

Outcome	HA + pps	No treat
Mean costs (€)	2,429	2,288
Mean LYs	20.0094	20.0094
Mean QALYs	18.9073	18.7132
NMB	305,099	302,082

Compared with no treatment	
Incremental costs (€)	137
Incremental QALYs	0.1786
ICUR (€/QALYs)	767
INMB	2,768

Compared with no treatment	
Incremental costs (€)	140
Incremental QALYs	0.1941
ICUR (€/QALYs)	723
INMB	3,017

Outcome	HA	No treat
Mean costs (€)	2,020	1,909
Mean LYs	18.4096	18.4096
Mean QALYs	17.3445	17.2284
NMB	280,089	278,311
**Compared with no treatment**		
Incremental costs (€)	110	
Incremental QALYs	0.1161	
ICUR (€/QALYs)	950	
INMB	1,778	

Outcome	HA	No treat
Mean costs (€)	2,405	2,288
Mean LYs	20.0094	20.0094
Mean QALYs	18.8416	18.7132
NMB	304,054	302,082
**Compared with no treatment**		
Incremental costs (€)	116	
Incremental QALYs	0.1284	
ICUR (€/QALYs)	907	
INMB	1,972	

No dropout, partial compliance

Gender: male

Outcome	HA + pps	HA
Mean costs (€)	2,563	2,460
Mean LYs	18.4096	18.4096
Mean QALYs	18.0006	17.7898
NMB	290,217	286,892
**Compared with HA w/out post-purchase service**		
Incremental costs (€)	103	

Gender: female

Outcome	HA + pps	HA
Mean costs (€)	2,957	2,846
Mean LYs	20.0094	20.0094
Mean QALYs	19.4988	19.2814
NMB	314,192	310,766
**Compared with HA w/out post-purchase service**		
Incremental costs (€)	111	

Incremental QALYs	0.2108
ICUR (€/QALYs)	490
INMB	3,325

Incremental QALYs	0.2174
ICUR (€/QALYs)	509
INMB	3,426

Outcome	HA + pps	No treat
Mean costs (€)	2,563	1,909
Mean LYs	18.4096	18.4096
Mean QALYs	18.0006	17.2284
NMB	290,217	278,311
**Compared with no treatment**		
Incremental costs (€)	654	
Incremental QALYs	0.7722	
ICUR (€/QALYs)	847	
INMB	11,906	

Outcome	HA + pps	No treat
Mean costs (€)	2,957	2,288
Mean LYs	20.0094	20.0094
Mean QALYs	19.4988	18.7132
NMB	314,192	302,082
**Compared with no treatment**		
Incremental costs (€)	668	
Incremental QALYs	0.7856	
ICUR (€/QALYs)	850	
INMB	12,110	

Outcome	HA	No treat
Mean costs (€)	2,460	1,909
Mean LYs	18.4096	18.4096
Mean QALYs	17.7898	17.2284
NMB	286,892	278,311
**Compared with no treatment**		
Incremental costs (€)	550	
Incremental QALYs	0.5614	
ICUR (€/QALYs)	980	
INMB	8,581	

Outcome	HA	No treat
Mean costs (€)	2,846	2,288
Mean LYs	20.0094	20.0094
Mean QALYs	19.2814	18.7132
NMB	310,766	302,082
**Compared with no treatment**		
Incremental costs (€)	558	
Incremental QALYs	0.5682	
ICUR (€/QALYs)	981	
INMB	8,685	

No dropout, full compliance

Gender: male

**Outcome**	**HA + pps**	**HA**
Mean costs (€)	2,580	2,472
Mean LYs	18.4096	18.4096
Mean QALYs	18.0301	17.8176
NMB	290,680	287,331
**Compared with HA w/out post-purchase service**		
Incremental costs (€)	107	
Incremental QALYs	0.2125	
ICUR (€/QALYs)	506	
INMB	3,350	

Gender: female

**Outcome**	**HA + pps**	**HA**
Mean costs (€)	2,976	2,859
Mean LYs	20.0094	20.0094
Mean QALYs	19.5321	19.3091
NMB	314,713	311,203
**Compared with HA w/out post-purchase service**		
Incremental costs (€)	117	
Incremental QALYs	0.2230	
ICUR (€/QALYs)	526	
INMB	3,510	

Gender: male

**Outcome**	**HA + pps**	**No treat**
Mean costs (€)	2,580	1,909
Mean LYs	18.4096	18.4096
Mean QALYs	18.0301	17.2284
NMB	290,680	278,311
**Compared with no treatment**		

Gender: female

**Outcome**	**HA + pps**	**No treat**
Mean costs (€)	2,976	2,288
Mean LYs	20.0094	20.0094
Mean QALYs	19.5321	18.7132
NMB	314,713	302,082
**Compared with no treatment**		

Incremental costs (€)	670		Incremental costs (€)	688
Incremental QALYs	0.8017		Incremental QALYs	0.8189
ICUR (€/QALYs)	836		ICUR (€/QALYs)	840
INMB	12,369		INMB	12,631

Outcome	HA	No treat
Mean costs (€)	2,472	1,909
Mean LYs	18.4096	18.4096
Mean QALYs	17.8176	17.2284
NMB	287,331	278,311
**Compared with no treatment**		
Incremental costs (€)	563	
Incremental QALYs	0.5891	
ICUR (€/QALYs)	955	
INMB	9,019	

Outcome	HA	No treat
Mean costs (€)	2,859	2,288
Mean LYs	20.0094	20.0094
Mean QALYs	19.3091	18.7132
NMB	311,203	302,082
**Compared with no treatment**		
Incremental costs (€)	570	
Incremental QALYs	0.5959	
ICUR (€/QALYs)	957	
INMB	9,121	

Public tariffs only, utilities Mandavia et al., 2020
Current dropout and compliance mix

Gender: male

Outcome	HA + pps	HA
Mean costs (€)	2,046	2,020
Mean LYs	18.4096	18.4096
Mean QALYs	17.5216	17.4926
NMB	282,943	282,498

Gender: female

Outcome	HA + pps	HA
Mean costs (€)	2,429	2,405
Mean LYs	20.0094	20.0094
Mean QALYs	19.0041	18.9712
NMB	306,674	306,162

Compared with HA w/out post-purchase service	
Incremental costs (€)	27
Incremental QALYs	0.0290
ICUR (€/QALYs)	918
INMB	445

Compared with HA w/out post-purchase service	
Incremental costs (€)	24
Incremental QALYs	0.0329
ICUR (€/QALYs)	727
INMB	512

Outcome	HA + pps	No treat
Mean costs (€)	2,046	1,909
Mean LYs	18.4096	18.4096
Mean QALYs	17.5216	17.4394
NMB	282,943	281,742
**Compared with no treatment**		
Incremental costs (€)	137	
Incremental QALYs	0.0822	
ICUR (€/QALYs)	1,666	
INMB	1,201	

Outcome	HA + pps	No treat
Mean costs (€)	2,429	2,288
Mean LYs	20.0094	20.0094
Mean QALYs	19.0041	18.9084
NMB	306673.6	305,257
**Compared with no treatment**		
Incremental costs (€)	140	
Incremental QALYs	0.0957	
ICUR (€/QALYs)	1,467	
INMB	1,416	

Outcome	HA	No treat
Mean costs (€)	2,020	1,909
Mean LYs	18.4096	18.4096
Mean QALYs	17.4926	17.4394
NMB	282,498	281,742
**Compared with no treatment**		
Incremental costs (€)	110	
Incremental QALYs	0.0532	

Outcome	HA	No treat
Mean costs (€)	2,405	2,288
Mean LYs	20.0094	20.0094
Mean QALYs	18.9712	18.9084
NMB	306,162	305,257
**Compared with no treatment**		
Incremental costs (€)	116	
Incremental QALYs	0.0628	

ICUR (€/QALYs)	2,074	ICUR (€/QALYs)	1,855
INMB	755	INMB	904

No dropout, partial compliance

Gender: male

Outcome	HA + pps	HA
Mean costs (€)	2,563	2,460
Mean LYs	18.4096	18.4096
Mean QALYs	17.8372	17.7157
NMB	287,559	285,686
**Compared with HA w/out post-purchase service**		
Incremental costs (€)	103	
Incremental QALYs	0.1215	
ICUR (€/QALYs)	850	
INMB	1,874	

Gender: female

Outcome	HA + pps	HA
Mean costs (€)	2,957	2,846
Mean LYs	20.0094	20.0094
Mean QALYs	19.3530	19.2156
NMB	311,820	309,696
**Compared with HA w/out post-purchase service**		
Incremental costs (€)	111	
Incremental QALYs	0.1374	
ICUR (€/QALYs)	805	
INMB	2,124	

Outcome	HA + pps	No treat
Mean costs (€)	2,563	1,909
Mean LYs	18.4096	18.4096
Mean QALYs	17.8372	17.4394

Outcome	HA + pps	No treat
Mean costs (€)	2,957	2,288
Mean LYs	20.0094	20.0094
Mean QALYs	19.3530	18.9084

NMB	287,559	281,742
**Compared with no treatment**		
Incremental costs (€)	654	
Incremental QALYs	0.3978	
ICUR (€/QALYs)	1,643	
INMB	5,817	

NMB	311,820	305,257
**Compared with no treatment**		
Incremental costs (€)	668	
Incremental QALYs	0.4446	
ICUR (€/QALYs)	1,503	
INMB	6,563	

**Outcome**	**HA**	**No treat**
Mean costs (€)	2,460	1,909
Mean LYs	18.4096	18.4096
Mean QALYs	17.7157	17.4394
NMB	285,686	281,742
**Compared with no treatment**		
Incremental costs (€)	550	
Incremental QALYs	0.2763	
ICUR (€/QALYs)	1,992	
INMB	3,943	

**Outcome**	**HA**	**No treat**
Mean costs (€)	2,846	2,288
Mean LYs	20.0094	20.0094
Mean QALYs	19.2156	18.9084
NMB	309,696	305,257
**Compared with no treatment**		
Incremental costs (€)	558	
Incremental QALYs	0.3072	
ICUR (€/QALYs)	1,815	
INMB	4,439	

No dropout, full compliance

Gender: male

**Outcome**	**HA + pps**	**HA**
Mean costs (€)	2,580	2,472
Mean LYs	18.4096	18.4096
Mean QALYs	17.8562	17.7307
NMB	287,852	285,917

Gender: female

**Outcome**	**HA + pps**	**HA**
Mean costs (€)	2,976	2,859
Mean LYs	20.0094	20.0094
Mean QALYs	19.3756	19.2322
NMB	312,168	309,954

Compared with HA w/out post-purchase service	
Incremental costs (€)	107
Incremental QALYs	0.1255
ICUR (€/QALYs)	856
INMB	1,934

Compared with HA w/out post-purchase service	
Incremental costs (€)	117
Incremental QALYs	0.1434
ICUR (€/QALYs)	819
INMB	2214.657891

Outcome	HA + pps	No treat
Mean costs (€)	2,580	1,909
Mean LYs	18.4096	18.4096
Mean QALYs	17.8562	17.4394
NMB	287,852	281,742
**Compared with no treatment**		
Incremental costs (€)	670	
Incremental QALYs	0.4168	
ICUR (€/QALYs)	1,608	
INMB	6,109	

Outcome	HA + pps	No treat
Mean costs (€)	2,976	2,288
Mean LYs	20.0094	20.0094
Mean QALYs	19.3756	18.9084
NMB	312,168	305,257
**Compared with no treatment**		
Incremental costs (€)	688	
Incremental QALYs	0.4672	
ICUR (€/QALYs)	1,472	
INMB	6,911	

Outcome	HA	No treat
Mean costs (€)	2,472	1,909
Mean LYs	18.4096	18.4096
Mean QALYs	17.7307	17.4394
NMB	285,917	281,742
**Compared with no treatment**		
Incremental costs (€)	563	
Incremental QALYs	0.2913	

Outcome	HA	No treat
Mean costs (€)	2,859	2,288
Mean LYs	20.0094	20.0094
Mean QALYs	19.2322	18.9084
NMB	309,954	305,257
**Compared with no treatment**		
Incremental costs (€)	570	
Incremental QALYs	0.3238	

ICUR (€/QALYs)	1,932	ICUR (€/QALYs)	1,761
INMB	4,175	INMB	4,696

Public tariff for the device coinciding with private market price, utilities Shield 2018

Current dropout and compliance mix

Gender: male

**Outcome**	**HA + pps**	**HA**
Mean costs (€)	2,339	2,243
Mean LYs	18.4096	18.4096
Mean QALYs	17.4070	17.3445
NMB	280,787	279,866
**Compared with HA w/out post-purchase service**		
Incremental costs (€)	96	
Incremental QALYs	0.0625	
ICUR (€/QALYs)	1,531	
INMB	921	

Gender: female

**Outcome**	**HA + pps**	**HA**
Mean costs (€)	2,764	2,664
Mean LYs	20.0094	20.0094
Mean QALYs	18.9073	18.8416
NMB	304,764	303,795
**Compared with HA w/out post-purchase service**		
Incremental costs (€)	100	
Incremental QALYs	0.0657	
ICUR (€/QALYs)	1,521	
INMB	969	

Gender: male

**Outcome**	**HA + pps**	**No treat**
Mean costs (€)	2,339	1,909
Mean LYs	18.4096	18.4096

Gender: female

**Outcome**	**HA + pps**	**No treat**
Mean costs (€)	2,764	2,288
Mean LYs	20.0094	20.0094

Mean QALYs	17.4070	17.2284	Mean QALYs	18.9073	18.7132
NMB	280,787	278,311	NMB	304,764	302,082
**Compared with no treatment**			**Compared with no treatment**		
Incremental costs (€)	429		Incremental costs (€)	476	
Incremental QALYs	0.1786		Incremental QALYs	0.1941	
ICUR (€/QALYs)	2,404		ICUR (€/QALYs)	2,450	
INMB	2,476		INMB	2,682	

**Outcome**	**HA**	**No treat**	**Outcome**	**HA**	**No treat**
Mean costs (€)	2,243	1,909	Mean costs (€)	2,664	2,288
Mean LYs	18.4096	18.4096	Mean LYs	20.0094	20.0094
Mean QALYs	17.3445	17.2284	Mean QALYs	18.8416	18.7132
NMB	279,866	278,311	NMB	303,795	302,082
**Compared with no treatment**			**Compared with no treatment**		
Incremental costs (€)	334		Incremental costs (€)	376	
Incremental QALYs	0.1161		Incremental QALYs	0.1284	
ICUR (€/QALYs)	2,875		ICUR (€/QALYs)	2,925	
INMB	1,555		INMB	1,713	

No dropout, partial compliance

**Gender: male**			**Gender: female**		
**Outcome**	**HA + pps**	**HA**	**Outcome**	**HA + pps**	**HA**
Mean costs (€)	3,952	3,601	Mean costs (€)	4,478	4,076
Mean LYs	18.4096	18.4096	Mean LYs	20.0094	20.0094
Mean QALYs	18.0006	17.7898	Mean QALYs	19.4988	19.2814

NMB	288,828	285,751
**Compared with HA w/out post-purchase service**		
Incremental costs (€)	351	
Incremental QALYs	0.2108	
ICUR (€/QALYs)	1,667	
INMB	3,077	

**Outcome**	**HA + pps**	**No treat**
Mean costs (€)	3,952	1,909
Mean LYs	18.4096	18.4096
Mean QALYs	18.0006	17.2284
NMB	288,828	278,311
**Compared with no treatment**		
Incremental costs (€)	2,043	
Incremental QALYs	0.7722	
ICUR (€/QALYs)	2,646	
INMB	10,517	

**Outcome**	**HA**	**No treat**
Mean costs (€)	3,601	1,909
Mean LYs	18.4096	18.4096
Mean QALYs	17.7898	17.2284
NMB	285,751	278,311
**Compared with no treatment**		
Incremental costs (€)	1,692	

NMB	312,670	309,537
**Compared with HA w/out post-purchase service**		
Incremental costs (€)	403	
Incremental QALYs	0.2174	
ICUR (€/QALYs)	1,853	
INMB	3,133	

**Outcome**	**HA + pps**	**No treat**
Mean costs (€)	4,478	2,288
Mean LYs	20.0094	20.0094
Mean QALYs	19.4988	18.7132
NMB	312,670	302,082
**Compared with no treatment**		
Incremental costs (€)	2,190	
Incremental QALYs	0.7856	
ICUR (€/QALYs)	2,788	
INMB	10,588	

**Outcome**	**HA**	**No treat**
Mean costs (€)	4,076	2,288
Mean LYs	20.0094	20.0094
Mean QALYs	19.2814	18.7132
NMB	309,537	302,082
**Compared with no treatment**		
Incremental costs (€)	1,787	

Incremental QALYs	0.5614	Incremental QALYs	0.5682
ICUR (€/QALYs)	3,013	ICUR (€/QALYs)	3,145
INMB	7,440	INMB	7,455

No dropout, full compliance

Gender: male

Outcome	HA + pps	HA
Mean costs (€)	4,007	3,645
Mean LYs	18.4096	18.4096
Mean QALYs	18.0301	17.8176
NMB	289,253	286,158
**Compared with HA w/out post-purchase service**		
Incremental costs (€)	362	
Incremental QALYs	0.2125	
ICUR (€/QALYs)	1,703	
INMB	3,095	

Gender: female

Outcome	HA + pps	HA
Mean costs (€)	4,547	4,123
Mean LYs	20.0094	20.0094
Mean QALYs	19.5321	19.3091
NMB	313,142	309,939
**Compared with HA w/out post-purchase service**		
Incremental costs (€)	424	
Incremental QALYs	0.2230	
ICUR (€/QALYs)	1,900	
INMB	3,203	

Outcome	HA + pps	No treat
Mean costs (€)	4,007	1,909
Mean LYs	18.4096	18.4096

Outcome	HA + pps	No treat
Mean costs (€)	4,547	2,288
Mean LYs	20.0094	20.0094

Mean QALYs	18.0301	17.2284	Mean QALYs	19.5321	18.7132
NMB	289,253	278,311	NMB	313,142	302,082
**Compared with no treatment**			**Compared with no treatment**		
Incremental costs (€)	2,097		Incremental costs (€)	2,259	
Incremental QALYs	0.8017		Incremental QALYs	0.8189	
ICUR (€/QALYs)	2,616		ICUR (€/QALYs)	2,758	
INMB	10,942		INMB	11,060	

Outcome	HA	No treat	Outcome	HA	No treat
Mean costs (€)	3,645	1,909	Mean costs (€)	4,123	2,288
Mean LYs	18.4096	18.4096	Mean LYs	20.0094	20.0094
Mean QALYs	17.8176	17.2284	Mean QALYs	19.3091	18.7132
NMB	286,158	278,311	NMB	309938.7	302,082
**Compared with no treatment**			**Compared with no treatment**		
Incremental costs (€)	1,735		Incremental costs (€)	1,835	
Incremental QALYs	0.5891		Incremental QALYs	0.5959	
ICUR (€/QALYs)	2,946		ICUR (€/QALYs)	3,079	
INMB	7,847		INMB	7,857	

Public tariff for the device coinciding with private market price, utilities Mandavia et al., 2020

Current dropout and compliance mix

Gender: male | **Gender: female**

Outcome	HA + pps	HA	Outcome	HA + pps	HA
Mean costs (€)	2,339	2,243	Mean costs (€)	2,764	2,664
Mean LYs	18.4096	18.4096	Mean LYs	20.0094	20.0094

Mean QALYs	17.5216	17.4926
NMB	282,651	282,274
**Compared with HA w/out post-purchase service**		
Incremental costs (€)	96	
Incremental QALYs	0.0290	
ICUR (€/QALYs)	3,298	
INMB	376	

Mean QALYs	19.0041	18.9712
NMB	306,338	305,903
**Compared with HA w/out post-purchase service**		
Incremental costs (€)	100	
Incremental QALYs	0.0329	
ICUR (€/QALYs)	3,034	
INMB	436	

**Outcome**	**HA + pps**	**No treat**
Mean costs (€)	2,339	1,909
Mean LYs	18.4096	18.4096
Mean QALYs	17.5216	17.4394
NMB	282,651	281,742
**Compared with no treatment**		
Incremental costs (€)	429	
Incremental QALYs	0.0822	
ICUR (€/QALYs)	5,222	
INMB	908	

**Outcome**	**HA + pps**	**No treat**
Mean costs (€)	2,764	2,288
Mean LYs	20.0094	20.0094
Mean QALYs	19.0041	18.9084
NMB	306,338	305,257
**Compared with no treatment**		
Incremental costs (€)	476	
Incremental QALYs	0.0957	
ICUR (€/QALYs)	4,969	
INMB	1,081	

**Outcome**	**HA**	**No treat**
Mean costs (€)	2,243	1,909
Mean LYs	18.4096	18.4096
Mean QALYs	17.4926	17.4394
NMB	282,274	281,742
**Compared with no treatment**		

**Outcome**	**HA**	**No treat**
Mean costs (€)	2,664	2,288
Mean LYs	20.0094	20.0094
Mean QALYs	18.9712	18.9084
NMB	305,903	305,257
**Compared with no treatment**		

Incremental costs (€)	334		Incremental costs (€)	376
Incremental QALYs	0.0532		Incremental QALYs	0.0628
ICUR (€/QALYs)	6,272		ICUR (€/QALYs)	5,985
INMB	532		INMB	645

No dropout, partial compliance

Gender: male

Outcome	HA + pps	HA
Mean costs (€)	3,952	3,601
Mean LYs	18.4096	18.4096
Mean QALYs	17.8372	17.7157
NMB	286,170	284,544
**Compared with HA w/out post-purchase service**		
Incremental costs (€)	351	
Incremental QALYs	0.1215	
ICUR (€/QALYs)	2,890	
INMB	1,626	

Gender: female

Outcome	HA + pps	HA
Mean costs (€)	4,478	4,076
Mean LYs	20.0094	20.0094
Mean QALYs	19.3530	19.2156
NMB	310,299	308,467
**Compared with HA w/out post-purchase service**		
Incremental costs (€)	403	
Incremental QALYs	0.1374	
ICUR (€/QALYs)	2,931	
INMB	1,832	

Outcome	HA + pps	No treat	Outcome	HA + pps	No treat
Mean costs (€)	3,952	1,909	Mean costs (€)	4,478	2,288

Mean LYs	18.4096	18.4096	Mean LYs	20.0094	20.0094
Mean QALYs	17.8372	17.4394	Mean QALYs	19.3530	18.9084
NMB	286,170	281,742	NMB	310,299	305,257
**Compared with no treatment**			**Compared with no treatment**		
Incremental costs (€)	2,043		Incremental costs (€)	2,190	
Incremental QALYs	0.3978		Incremental QALYs	0.4446	
ICUR (€/QALYs)	5,136		ICUR (€/QALYs)	4,926	
INMB	4,427		INMB	5,041	

Outcome	HA	No treat	Outcome	HA	No treat
Mean costs (€)	3,601	1,909	Mean costs (€)	4,076	2,288
Mean LYs	18.4096	18.4096	Mean LYs	20.0094	20.0094
Mean QALYs	17.7157	17.4394	Mean QALYs	19.2156	18.9084
NMB	284,544	281,742	NMB	308,467	305,257
**Compared with no treatment**			**Compared with no treatment**		
Incremental costs (€)	1,692		Incremental costs (€)	1,787	
Incremental QALYs	0.2763		Incremental QALYs	0.3072	
ICUR (€/QALYs)	6,123		ICUR (€/QALYs)	5,818	
INMB	2,802		INMB	3,209	

No dropout, full compliance

**Gender: male**			**Gender: female**		
**Outcome**	**HA + pps**	**HA**	**Outcome**	**HA + pps**	**HA**
Mean costs (€)	4,007	3,645	Mean costs (€)	4,547	4,123

Mean LYs	18.4096	18.4096
Mean QALYs	17.8562	17.7307
NMB	286,425	284,745
**Compared with HA w/out post-purchase service**		
Incremental costs (€)	362	
Incremental QALYs	0.1255	
ICUR (€/QALYs)	2,884	
INMB	1,680	

**Outcome**	**HA + pps**	**No treat**
Mean costs (€)	4,007	1,909
Mean LYs	18.4096	18.4096
Mean QALYs	17.8562	17.4394
NMB	286,425	281,742
**Compared with no treatment**		
Incremental costs (€)	2,097	
Incremental QALYs	0.4168	
ICUR (€/QALYs)	5,032	
INMB	4,682	

**Outcome**	**HA**	**No treat**
Mean costs (€)	3,645	1,909
Mean LYs	18.4096	18.4096
Mean QALYs	17.7307	17.4394
NMB	284,745	281,742

Mean LYs	20.0094	20.0094
Mean QALYs	19.3756	19.2322
NMB	310,597	308,689
**Compared with HA w/out post-purchase service**		
Incremental costs (€)	424	
Incremental QALYs	0.1434	
ICUR (€/QALYs)	2,956	
INMB	1,908	

**Outcome**	**HA + pps**	**No treat**
Mean costs (€)	4,547	2,288
Mean LYs	20.0094	20.0094
Mean QALYs	19.3756	18.9084
NMB	310,597	305,257
**Compared with no treatment**		
Incremental costs (€)	2,259	
Incremental QALYs	0.4672	
ICUR (€/QALYs)	4,835	
INMB	5,340	

**Outcome**	**HA**	**No treat**
Mean costs (€)	4,123	2,288
Mean LYs	20.0094	20.0094
Mean QALYs	19.2322	18.9084
NMB	308,689	305,257

Compared with no treatment	
Incremental costs (€)	1,735
Incremental QALYs	0.2913
ICUR (€/QALYs)	5,958
INMB	3,002

Compared with no treatment	
Incremental costs (€)	1,835
Incremental QALYs	0.3238
ICUR (€/QALYs)	5,666
INMB	3,432

CHEERS checklist

Table 8 CHEERS checklist—Items to include when reporting economic evaluations of health interventions

Section/item	Item no.	Recommendation	Page, lines
**Title and abstract**			
Title	1	Identify the study as an economic evaluation or use more specific terms such as "cost-effectiveness analysis", and describe the interventions compared.	Title page
Abstract	2	Provide a structured summary of objectives, perspective, setting, methods (including study design and inputs), results (including base case and uncertainty analyses), and conclusions.	p. 1, ll. 4-23
**Introduction**			
Background and objectives	3	Provide an explicit statement of the broader context for the study.	pp. 2-3, ll. 27-53
		Present the study question and its relevance for health policy or practice decisions.	p. 3, ll. 54-56
**Methods**			
Target population and subgroups	4	Describe characteristics of the base case population and subgroups analysed, including why they were chosen.	p. 3, ll. 60, 72-78
Setting and location	5	State relevant aspects of the system(s) in which the decision(s) need(s) to be made.	p. 3, ll. 56, 63-66
Study perspective	6	Describe the perspective of the study and relate this to the costs being evaluated.	p. 3, ll. 66-68
Comparators	7	Describe the interventions or strategies being compared and state why they were chosen.	p. 3, ll. 58-59
Time horizon	8	State the time horizon(s) over which costs and consequences are being evaluated and say why appropriate.	p. 3, ll. 72-74
Discount rate	9	Report the choice of discount rate(s) used for costs and outcomes and say why appropriate.	p. 3, ll. 68-69
Choice of health outcomes	10	Describe what outcomes were used as the measure(s) of benefit in the evaluation and their relevance for the type of analysis performed.	p. 3, 60-66
	11°	*Single study-based estimates:* Describe fully the design features of the single effectiveness study and	N/A

Section/item	Item no.	Recommendation	Page, lines
Measurement of effectiveness		why the single study was a sufficient source of clinical effectiveness data.	
	11b	*Synthesis-based estimates*: Describe fully the methods used for identification of included studies and synthesis of clinical effectiveness data.	pp. 5-6, ll. 106-148
Measurement and valuation of preference based outcomes	12	If applicable, describe the population and methods used to elicit preferences for outcomes.	pp. 6-7, ll. 150-165
Estimating resources and costs	13°	*Single study-based economic evaluation:* Describe approaches used to estimate resource use associated with the alternative interventions. Describe primary or secondary research methods for valuing each resource item in terms of its unit cost. Describe any adjustments made to approximate to opportunity costs.	N/A
	13b	*Model-based economic evaluation:* Describe approaches and data sources used to estimate resource use associated with model health states. Describe primary or secondary research methods for valuing each resource item in terms of its unit cost. Describe any adjustments made to approximate to opportunity costs.	pp. 7-9, ll. 167-222
Currency, price date, and conversion	14	Report the dates of the estimated resource quantities and unit costs. Describe methods for adjusting estimated unit costs to the year of reported costs if necessary. Describe methods for converting costs into a common currency base and the exchange rate.	p. 9, l. 222
Choice of model	15	Describe and give reasons for the specific type of decision-analytical model used. Providing a figure to show model structure is strongly recommended.	pp. 3-4, ll. 72-102
Assumptions	16	Describe all structural or other assumptions underpinning the decision-analytical model.	pp. 3-4, ll. 72-102
Analytical methods	17	Describe all analytical methods supporting the evaluation. This could include methods for dealing with skewed, missing, or censored data; extrapolation methods; methods for pooling data; approaches to validate or make adjustments (such as half cycle corrections) to a model; and methods for handling population heterogeneity and uncertainty.	p. 5, ll. 106-121; pp. 9-10, ll. 226-239
**Results**			
Study parameters	18	Report the values, ranges, references, and, if used, probability distributions for all parameters. Report reasons or sources for distributions used to represent	Table 1

Section/item	Item no.	Recommendation	Page, lines
		uncertainty where appropriate. Providing a table to show the input values is strongly recommended.	
Incremental costs and outcomes	19	For each intervention, report mean values for the main categories of estimated costs and outcomes of interest, as well as mean differences between the comparator groups. If applicable, report incremental cost-effectiveness ratios.	Table 2; pp. 10, ll. 243-257
Characterising uncertainty	20a	*Single study-based economic evaluation:* Describe the effects of sampling uncertainty for the estimated incremental cost and incremental effectiveness parameters, together with the impact of methodological assumptions (such as discount rate, study perspective).	N/A
	20b	*Model-based economic evaluation:* Describe the effects on the results of uncertainty for all input parameters, and uncertainty related to the structure of the model and assumptions.	p. 11, ll. 259-265
Characterising heterogeneity	21	If applicable, report differences in costs, outcomes, or cost-effectiveness that can be explained by variations between subgroups of patients with different baseline characteristics or other observed variability in effects that are not reducible by more information.	N/A
**Discussion**			
Study findings, limitations, generalisability, and current knowledge	22	Summarise key study findings and describe how they support the conclusions reached. Discuss limitations and the generalisability of the findings and how the findings fit with current knowledge.	pp. 11-12, ll. 270-304
**Other**			
Source of funding	23	Describe how the study was funded and the role of the funder in the identification, design, conduct, and reporting of the analysis. Describe other non-monetary sources of support.	Title page
Conflicts of interest	24	Describe any potential for conflict of interest of study contributors in accordance with journal policy. In the absence of a journal policy, we recommend authors comply with International Committee of Medical Journal Editors recommendations.	Title page

For consistency, the CHEERS statement checklist format is based on the format of the CONSORT statement checklist

References

1. Homans NC, Metselaar RM, Dingemanse JG, et al. Prevalence of age-related hearing loss, including sex differences, in older adults in a large cohort study. *The Laryngoscope.* 2017;127(3):725-730.
2. Boas G, Van Der Stel H, Peters H, Joore M, Anteunis L. DYNAMIC MODELING IN MEDICAL TECHNOLOGY ASSESSMENT. *International journal of technology assessment in health care.* 2001;17(4):618.
3. Joore MA, van der Stel H, Peters HJ, Boas GM, Anteunis LJ. The cost-effectiveness of hearing-aid fitting in the Netherlands. *Archives of Otolaryngology–Head & Neck Surgery.* 2003;129(3):297-304.
4. Chao T-K, Chen TH-H. Cost-effectiveness of hearing aids in the hearing-impaired elderly: a probabilistic approach. *Otology & Neurotology.* 2008;29(6):776-783.
5. Mandavia R, Horstink YM, Grutters JP, et al. The Potential Added Value of Novel Hearing Therapeutics: An Early Health Economic Model for Hearing Loss. *Otology & Neurotology.* 2020;41(8):1033-1041.
6. Anovum. *EuroTrak Italy 2018.* 2018.
7. CENSIS. *Sentirsi bene. Il valore sociale dell'audioprotesi.* 2019.
8. Shield B. Hearing Loss – Numbers and Costs. Evaluation of the social and economic costs of hearing impairment. A report for Hear-It AISBL. *Hear-It AISBL.* 2018:1-250.
9. Barton GR, Bankart J, Davis AC, Summerfield QA. Comparing Utility Scores Before and After Hearing-Aid Provision. Results According to the EQ-5D, HUI3 and SF-6D. *Appl Health Econ Health Policy.* 2004;3(2):103-105.
10. Davis A, Smith P, Ferguson M, Stephens D, Gianopoulos I. Acceptability, benefit and costs of early screening for hearing disability: a study of potential screening tests and models. *Health Technology Assessment.* 2007;11(42).
11. Swan I, Guy F, Akeroyd M. Health-related quality of life before and after management in adults referred to otolaryngology: a prospective national study. *Clinical Otolaryngology.* 2012;37(1):35-43.
12. Linssen A, Joore M, Theunissen E, Anteunis L. The effects and costs of a hearing screening and rehabilitation program in residential care homes for the elderly in the Netherlands1. *American Journal of Audiology.* 2013.
13. Arndt S, Aschendorff A, Laszig R, et al. Comparison of pseudobinaural hearing to real binaural hearing rehabilitation after cochlear implantation in patients with unilateral deafness and tinnitus. *Otology & neurotology.* 2011;32(1):39-47.
14. Ministero della Sanità. Nomenclatore dell'assistenza specialistica ambulatoriale. 2017.
15. CEIS. *Acquisizione di apparecchi acustici da parte del Sistema Sanitario Nazionale: Alcune considerazioni sulla transizione da un sistema tariffario (D.M. 332/1999) ad un sistema basato sulle procedure pubbliche di acquisto (D.P.C.M. 12/01/2017).* CEIS Stat Brief N. 22020.
16. Ministero della Sanità. Decreto 27 agosto 1999 , n. 332 Regolamento recante norme per le prestazioni di assistenza protesica erogabili nell'ambito del Servizio sanitario nazionale: modalita' di erogazione e tariffe. G.U. Serie Generale , n. 227 del 27 settembre 19991999.
17. Scuffham P, Chaplin S, Legood R. Incidence and costs of unintentional falls in older people in the United Kingdom. *Journal of Epidemiology & Community Health.* 2003;57(9):740-744.
18. Girard SA, Leroux T, Verreault R, et al. Falls risk and hospitalization among retired workers with occupational noise-induced hearing loss. *Canadian Journal on Aging/La Revue canadienne du vieillissement.* 2014;33(1):84-91.
19. Mahmoudi E, Basu T, Langa K, et al. Can hearing aids delay time to diagnosis of dementia, depression, or falls in older adults? *Journal of the American Geriatrics Society.* 2019;67(11):2362-2369.

20. McDaid D, Park A-L, Chadha S. Estimating the global costs of hearing loss. *International Journal of Audiology.* 2021;60(3):162-170.
21. ILOSTAT. Unemployment Rates and Employment to Population Ratios. 2020; www.ilo.org/ilostat.
22. Nachtegaal J, Heymans MW, van Tulder MW, Goverts ST, Festen JM, Kramer SE. Comparing health care use and related costs between groups with and without hearing impairment. *International journal of audiology.* 2010;49(12):881-890.
23. Kochkin S. MarkeTrak VIII: The efficacy of hearing aids in achieving compensation equity in the workplace. *The Hearing Journal.* 2010;63(10):19-28.
